# Altered immune cell profiles in blood of mature/peripheral T-cell leukemia/lymphoma patients: an EuroFlow study

**DOI:** 10.3389/fimmu.2025.1561152

**Published:** 2025-03-21

**Authors:** F. Javier Morán-Plata, Noemí Muñoz-García, Susana Barrena, Ana Yeguas, Ana Balanzategui, Sonia Carretero-Domínguez, Quentin Lécrevisse, María González-González, Sheila Mateos, Lidia Silos, Miguel Alcoceba, Fernando Solano, Miriam López-Parra, Vitor Botafogo, Alberto Orfao, Julia Almeida

**Affiliations:** ^1^ Translational and Clinical Research Program, Cancer Research Center (IBMCC, CSIC – University of Salamanca), Cytometry Service, NUCLEUS, Department of Medicine, University of Salamanca (Departamento de Medicina, Universidad de Salamanca), Salamanca, Spain; ^2^ Institute of Biomedical Research of Salamanca (IBSAL), Salamanca, Spain; ^3^ Service of Hematology, University Hospital of Salamanca, Salamanca, Spain; ^4^ Biomedical Research Networking Centre Consortium of Oncology (CIBERONC), Instituto de Salud Carlos III, Madrid, Spain; ^5^ Hospital Ntra Sra del Prado, Talavera De La Reina, Spain

**Keywords:** mature/peripheral T-cell leukemia/lymphoma, T-prolymphocytic leukemia, Sézary syndrome/mycosis fungoides, T-large granular lymphocytic leukemia, tumor-associated immune cell profiles, blood immune cells

## Abstract

**Introduction:**

The interactions between T-cell chronic lymphoproliferative disorder (T-CLPD) tumor cells and the bystander immune cells may play a critical role in the failure of immune surveillance and disease progression, but the altered blood immune profiles of T-CLPD remain unknown.

**Methods:**

Here we analyzed the distribution of residual non-tumoral immune cells in blood of 47 T-CLPD patients -14 T-prolymphocytic leukemia (T-PLL), 7 Sézary syndrome/mycosis fungoides (SS/MF) and 26 T-large granular lymphocytic leukemia (T-LGLL)-, as tumor models of neoplastic T-cells that resemble naive/central memory (N/CM), memory and terminal effector T-cells, respectively, compared to 110 age- and sex-matched healthy donors, using spectral flow cytometry.

**Results:**

Overall, our results showed deeply altered immune cell profiles in T-PLL, characterized by significantly increased counts of monocytes, dendritic cells, B-cells, NK-cells and innate lymphoid cells (ILC) -particularly ILC3-, together with reduced normal T-cells. In contrast, SS/MF showed neutrophilia, associated with decreased numbers of dendritic cells and NK-cells, potentially reflecting their increased migration from blood to the skin. In turn, T-LGLL displayed the mildest immune impairment, dependent on the TCD4+ *vs*. TCD8+ nature of the clonal T-cells and presence of *STAT3* mutations among TαβCD8+ T-LGLL cases. Further dissection of the normal T-cell compartment showed a significant reduction of the earliest T-cell maturation compartments (N/CM) in T-PLL and SS/MF, whereas T-cells remained within normal ranges in T-LGLL, with only a minor reduction of N/CM T-cells.

**Conclusion:**

These findings point out the existence of differentially altered innate and adaptive immune cell profiles in the distinct diagnostic subtypes of T-CLPD, with progressively less pronounced alterations from T-PLL and SS/MF to T-LGLL.

## Introduction

The immune system plays a critical role in tumor immunosurveillance (1). Thus, it has been postulated that initially, tumor cells are *eliminated* by the immune system before becoming clinically detectable, followed by a phase of *equilibrium* during which less immunogenic tumor cell variants are selected, and finally, an *escape* phase occurs, where tumor cells evade immunosurveillance (2). Consequently, tumor progression not only involves the loss of immunosurveillance, but also results in a state of immunodeficiency (3, 4), which on its own, has been associated with an increased incidence of cancer (5, 6), such as in primary and secondary immunodeficiency patients (5, 6) including transplanted patients undergoing long-term immunosuppressive therapy (7, 8). Based on the proven role of the immune system in general, and of the immune cell components of the tumor microenvironment in particular (9), cancer cell-targeted immunotherapy, including treatments that modify the patient’s immune response to achieve therapeutic benefits, have become prominent nowadays in clinical oncology (10, 11).

Mature T-cell neoplasms or T-cell chronic lymphoproliferative disorders (T-CLPD) account for 10-15% of non-Hodgkin lymphomas in Western countries (12–14). They represent a diverse group of rare malignancies characterized by a high clinical, biological, phenotypic and genomic heterogeneity, often reflecting the specific location, functional properties and other biological features of normal reactive T-cells at specific functional and maturation-associated stages from which they are postulated to derive from (12–14). Thus, T-prolymphocytic leukemia (T-PLL) malignant cells show a typical phenotype that overlaps with that of naive/central memory (CM) T-cells with a variable functional (e.g., Th1 and/or Th17 (15–17)) profile, whereas in Sézary syndrome (SS)-mycosis fungoides (MF) and other T-CLPD (e.g., nodal follicular helper T-cell lymphoma and adult T-cell leukemia/lymphoma) neoplastic cells mimic a memory T-cell stage with a Th2 or Th17 (18, 19) (and TFH (20, 21) and Treg (22)) functional profile, respectively. In turn, in hepatosplenic T-cell lymphoma, neoplastic cells show a non-activated cytotoxic T-cell phenotype, corresponding to functionally immature (usually TCRγδ+) T-cells (14), while in T-large granular lymphocytic leukemia (T-LGLL) tumor cells mostly resemble terminal effector cytotoxic T-cells (23–25). Abnormal expansions of these functional subtypes of neoplastic T-cells accumulated at diverse stages of maturation might exert variable immunomodulatory effects on the bystander immune cells, including normal T-cells. Despite this, our current understanding about the tumor microenvironment in mature T-cell neoplasms (and its potential role in future immune-based treatments) still remains very limited (26).

Here, we investigated the distribution at diagnosis of normal blood immune cells in patients with three different subtypes of T-CLPD (i.e., T-PLL, SS/MF and T-LGLL) with a preferential focus on the analysis of the altered maturation-related and functionally-associated subsets of residual TCD4+ cells. Our ultimate goal was to gain insight into the altered immune cell profiles in blood of T-CLPD patients using these three diagnostic subtypes of the disease as models of mature T-cell malignancies derived from the earliest, intermediate and more mature T-cell compartments, respectively.

## Materials and methods

### Patients, controls and samples

A total of 157 peripheral blood (PB) samples from 47 patients diagnosed with mature/peripheral T-cell neoplasms [24 men and 23 women, with a median (range) age of 58 (19y - 90y) years (y)], and 110 age- and sex-matched healthy donors (HD) [63 men and 47 women, with a median age of 58y (range: 18y to 89y)] were studied. In every patient, PB samples were collected at diagnosis, in the absence of any previous or concomitant immunomodulatory treatment. Diagnosis and classification of T-cell neoplasms were retrospectively updated according to the WHO-2022 criteria (12), including T-LGLL (n=26), T-PLL (n=14) and SS/MF (n=7). Relevant clinical data about the disease behavior during follow-up were collected for the individual patients, including data on response to treatment –according to well-established criteria for each of the T-CLPD categories (27–29) – and disease progression, to analyze the potential association of these prognostic-related data with the immune profiles.

Every participant enrolled in this study gave his/her written informed consent to use their samples and the associated data, according to the Declaration of Helsinki, after the study had been approved by the local institutional Ethics Committee of the University Hospital of Salamanca/IBSAL (reference code: CEIm-2020/12/643C).

### Immunophenotypic studies

For flow cytometry studies, freshly collected (<24 hours) EDTA-anticoagulated whole PB samples, preserved at 4 °C until processing, were stained using the EuroFlow standard operating procedures (SOP) for staining of cell-surface markers alone or in combination with intracellular proteins, depending on the specific antibody panel used (**Supplementary Tables 1**, **2**), available at www.euroflow.org. After the appropriate antibody panels were applied for the diagnosis and classification of T-CLPD into the distinct WHO-2022 categories (**Supplementary Table 1**), patient samples were then stained using the EuroFlow immune-monitoring TCD4+ Tube (30) as backbone, to which additional antibodies were added for the unequivocal discrimination between tumor T-cells and normal residual T lymphocytes coexisting in the same sample. Those additional markers included CD2 and CD7 for the detection of leukemia/lymphoma-associated immunophenotypes (LAIP), anti-TRBC1 for the assessment of T-cell clonality, and CD8 (**Supplementary Table 2**).

Once the blood cells had been stained, samples were immediately measured in either an LSR-Fortessa X-20 flow cytometer -Becton/Dickinson Biosciences (BD) San Jose, CA- equipped with the FACSDiva™ (BD) software or a 5-laser Cytek^®^ Aurora spectral flow cytometer (Cytek Biosciences, Fremont, CA) using the SpectroFlo^®^ (Cytek Biosciences) software program. In both cases, the EuroFlow SOP for instrument setup and data acquisition were strictly followed (31). For data analysis, the INFINICYT™ software (Cytognos/BD, Salamanca, Spain) was used. In the patient samples, tumor T-cells were first identified and excluded from subsequent analyses as detailed in **Supplementary Material**. Afterward, normal residual leukocytes from both the patient and the HD samples were classified into the major leukocyte subsets, and TCD4+ lymphocytes subdivided into their multiple functionally-associated and maturation-related subsets, following previously reported analysis strategies, described in more detail in the **Supplementary Material** (30, 32). The functional validation of the chemokine receptor-based phenotypes used to identify the different Th-profiles of normal T-cells had been previously performed and reported by our group based on both *in vitro* cytokine secretion (assessed using an intracellular cytokine production assay by flow cytometry) and gene expression profile analyses (30). For each cell population, results were expressed as absolute number of cells/µL normalized to the age-matched HD values, as described elsewhere (33).

### Classification of T-PLL, SS/MF and T-LGLL cases according to the maturation-related and Th-associated phenotypic profiles of tumor T-cells

Patients within each T-CLPD diagnostic category were stratified according to the maturation and/or functional (Th-related) phenotypic profiles of their tumor T-cells, according to gating strategies for subsetting followed for normal T-cell populations (**Supplementary Material**). Thus, T-PLL cases were categorized into two main groups based on the maturation stage of tumor cells: i) -naive-naive/central memory (CM) and ii) CM-transitional memory (TM) T-PLL cells, and by the number of clonal cell populations displaying different Th-related phenotypes (cases with one *vs*. ≥2 phenotypes). Likewise, SS/MF were grouped according to the presence of Th2- *vs*. Th17-associated functional phenotypic profiles, whereas T-LGLL cases were stratified based on their TαβCD4+ *vs*. TαβCD8+ LGLL cytotoxic nature, the latter cases being further subdivided according to their specific Th-associated profile into a classical Th1 (i.e., CD183+ in the absence of other chemokine receptors) *vs*. an atypical Th1 profile lacking expression of any chemokine receptor, including CD183 (CR-).

### Assessment of T-cell clonality and molecular/genetic characterization of T-LGLL and T-PLL patients

The clonal nature of the abnormal/aberrant T-cell populations identified in the individual cases were confirmed, following well-established molecular approaches (12, 13, 34–37), which are briefly described in **Supplementary Material**. Analysis of *STAT3* and *STAT5b* gene mutations or evaluation of genetic alterations involving the *TCL1* (T-cell leukemia/lymphoma1) family of genes were performed on purified (FACS-sorted) clonal T-cells from individual T-LGLL and T-PLL cases, respectively (12, 13, 36, 37) (**Supplementary Material**).

### Statistical methods

Statistical significance of differences observed between groups was assessed with the χ² test or the Fisher’s exact test, for categorical variables, and with the Kruskal-Wallis or Mann-Whitney U tests for comparison of >2 or 2 continuous variables, respectively. Corrected (Benjamini–Hochberg procedure) *p*-values of ≤0.05 for multiple comparisons with a false discovery rate (FDR) of <10% were used to define statistically significant differences across the different lineages and diagnoses. Statistical analyses and graphical representations were carried out using the IBM-SPSS v28.0 (IBM, Armonk, NY) and GraphPad Prism V8 (GraphPad Software, San Diego, CA) software packages. P-values ≤0.05 were considered to be associated with statistical significance. Age-based normalization of leukocyte values was performed using the MIDAS software (BD/Cytognos SL, Salamanca, Spain), as previously described (33).

## Results

### Distribution of the major subsets of leukocytes in blood of patients with T-PLL, SS/MF and T-LGLL

**Figure 1:**
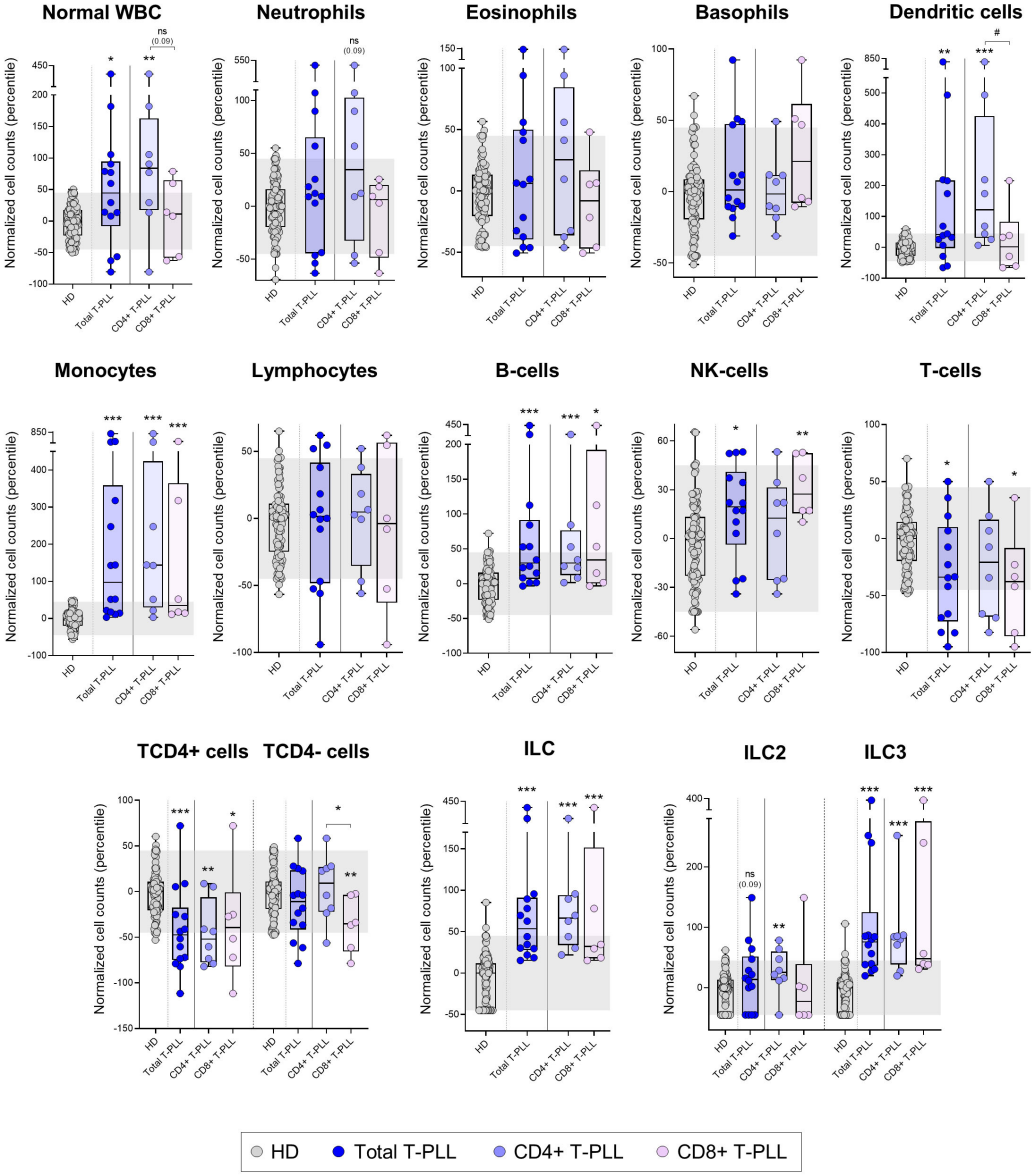
Distribution of the major normal/residual PB leukocyte subsets in blood of T-PLL patients and their T-PLL subgroups defined by the expression of CD4 *vs*. CD8 T-cell markers on the tumor cells. Box plots show the distribution of normalized counts of WBC and their major subsets in blood (in terms of percentile values relative to age-matched HD reference ranges) of total T-PLL, TCD4+ T-PLL and TCD8+ T-PLL patients. In all graphs, dots correspond to individual samples, while notched boxes represent the 25^th^ and 75^th^ percentile values; the lines inside the box correspond to median values (50^th^ percentile=0) and whiskers represent minimum and maximum values. Gray dots represent samples collected from HD, dark blue dots denote total T-PLL cases, while light blue and light violet dots correspond to the TCD4+ and TCD8+ (these latter including CD4-CD8+ and CD4+CD8+ cases) T-PLL subgroups, respectively. *p-value ≤0.05, **p-value ≤0.01 and ***p-value ≤0.001 *vs*. HD. Trend versus HD or between T-PLL groups is shown as p-values <0.1. P-values resulting from comparisons between the two T-PLL lineages are depicted as ^#^p-value ≤0.05. The gray shading corresponds to the 5^th^-95^th^ percentiles (values -45 and 45, respectively). (alphabetical order): HD, healthy donor; ILC, innate lymphoid cell; NK, natural-killer; ns, statistically not significant; T-PLL, T-prolymphocytic leukemia; WBC, white blood cell.

After exclusion of the tumor cells present in the sample, we observed significantly increased overall white blood cell (WBC) counts above normal HD values in T-PLL (p=0.011). This was due to significantly higher monocyte (p<0.001), dendritic cell (p=0.003), B-cell (p<0.001), NK-cell (p=0.012) and innate lymphoid cell (ILC) (p<0.001) counts. Of note, the increased absolute counts of monocytes, NK-cells and ILC2 was significantly higher (p ≤ 0.05) among T-PLL patients who progressed (n=8) *vs*. those who did not show disease progression (n=4) after a median (range) follow-up of 4.5 (2–6) years (**Supplementary Figure 1**). In contrast, no significant differences were found between T-PLL patients who reached complete remission (n=4) or showed partial remission (n=3) to therapy *vs*. patients with stable response disease/progression after first-line treatment (n=4) (data not shown). In turn, normal T-cells were found to be significantly reduced in T-PLL (p=0.019 *vs*. HD, particularly at the expense of lower TCD4^+^ cell counts (p<0.001) (**Figure 1**, **Supplementary Table 3**), without significant differences between patients who showed disease progression and those who did not progress (1270 T-cells/µL and 611 TCD4+ cells/µL *vs*. 1041 T-cells/µL and 451 TCD4+ cells/µL, respectively; p>0.05). These findings were consistently confirmed when T-PLL patients were broken down into the major TCD4+ T-PLL subgroup and, to a lesser extent also, the other less represented TCD8+ (CD4 positive or negative) T-PLL cases, except for normal WBC, NK-cell, total T-cell, TCD4- cytotoxic-cell and ILC2 counts in the latter TCD8+ T-PLL subgroup. Of note, T-PLL patients with a naive/naive-CM tumor cell phenotype showed a more pronounced increase in blood of both dendritic cells and NK-cells (p=0.002 and p=0.012, respectively) together with a greater decrease in total T-cells, and particularly TCD4+ cells (p=0.003 and p<0.001, respectively), compared to those T-PLL patients with a CM-TM profile (**Supplementary Figure 2**). In addition, patients whose tumor cells exhibited ≥2 different Th-associated phenotypes showed higher normal WBC (p=0.045), dendritic cell (p=0.001) and ILC2 cell (p=0.006) counts compared to those whose tumor cells expressed a more homogeneous single Th-associated phenotype (**Supplementary Figure 2**).

**Figure 2:**
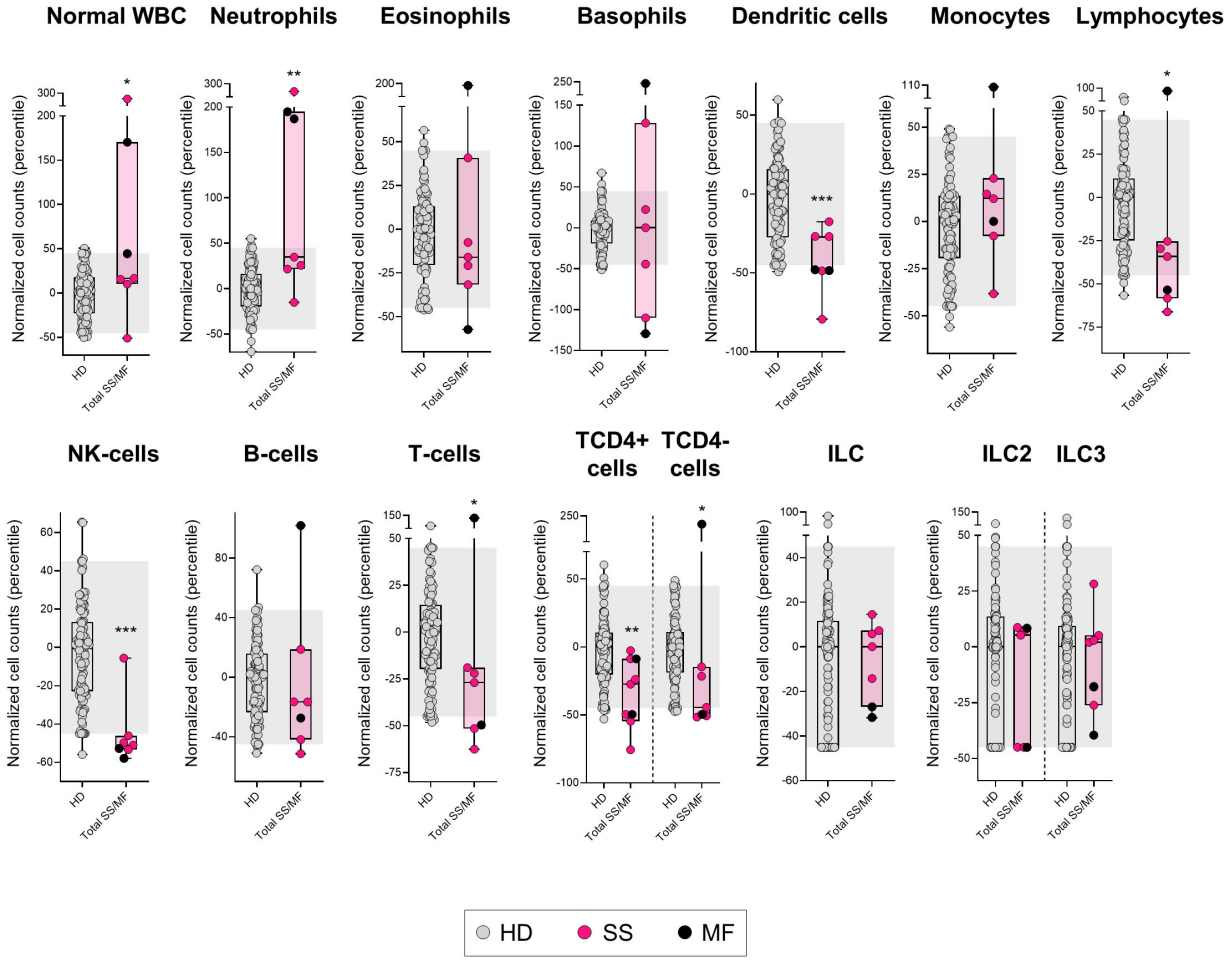
Distribution of the major normal/residual PB leukocyte subsets in in blood of SS/MF patients. Box plots show the distribution of normalized counts of WBC subsets in blood (percentile values relative to age-matched HD reference ranges) of SS/MF patients. In all plots, dots correspond to individual samples, while notched boxes represent the 25^th^ and 75^th^ percentile values; the lines inside the box correspond to median values (50^th^ percentile=0) and whiskers represent minimum and maximum values. Gray dots represent samples collected from HD, pink dots denote SS cases, while black dots correspond to MF. *p-value ≤0.05, **p-value ≤0.01 and ***p-value ≤0.001 *vs*. HD. The gray shading corresponds to the 5^th^-95^th^ percentiles (values -45 and 45, respectively). (alphabetical order): HD, healthy donor; ILC, innate lymphoid cell; MF, mycosis fungoides; NK, natural-killer; SS, Sézary syndrome; WBC, white blood cell.

Like T-PLL patients, SS/MF cases also showed an increased normal WBC count (p=0.048 *vs*. HD), but in association with a different pattern of alteration of the major blood leukocyte populations from that observed in T-PLL. Thus, SS/MF cases displayed significantly increased numbers of neutrophils in blood (p=0.003) associated with reduced dendritic cell (p=0.001) and total lymphocyte (p=0.015) counts, at the expense of lower NK-cell (p<0.001) and T-cell (p=0.020) (both TCD4+ and TCD4- cells; p=0.004 and p=0.016, respectively) numbers (**Figure 2**, **Supplementary Table 4**). Of note, increased neutrophil counts (p=0.014), together with decreased total lymphocyte (p=0.022), and particularly T-cell (p=0.037) numbers, were more pronounced in SS/MF cases with a Th17 *vs*. Th2 phenotypic profile of tumor cells, whereas the decrease in dendritic cell numbers in blood was more closely associated (p=0.004) with SS/MF cases with a Th2-associated tumor cell phenotype (**Supplementary Figure 3**). Of note, no significant association was found between these immune profiles and prognostic-related clinical data, such as response to treatment or disease progression, probably due to the limited number of cases (5/6 SS/MF patients were treated with systemic cytotoxic drugs, from which 4/5 progressed in a short period of time and 1/5 patient died at an early stage of therapy due to treatment toxicity).

**Figure 3:**
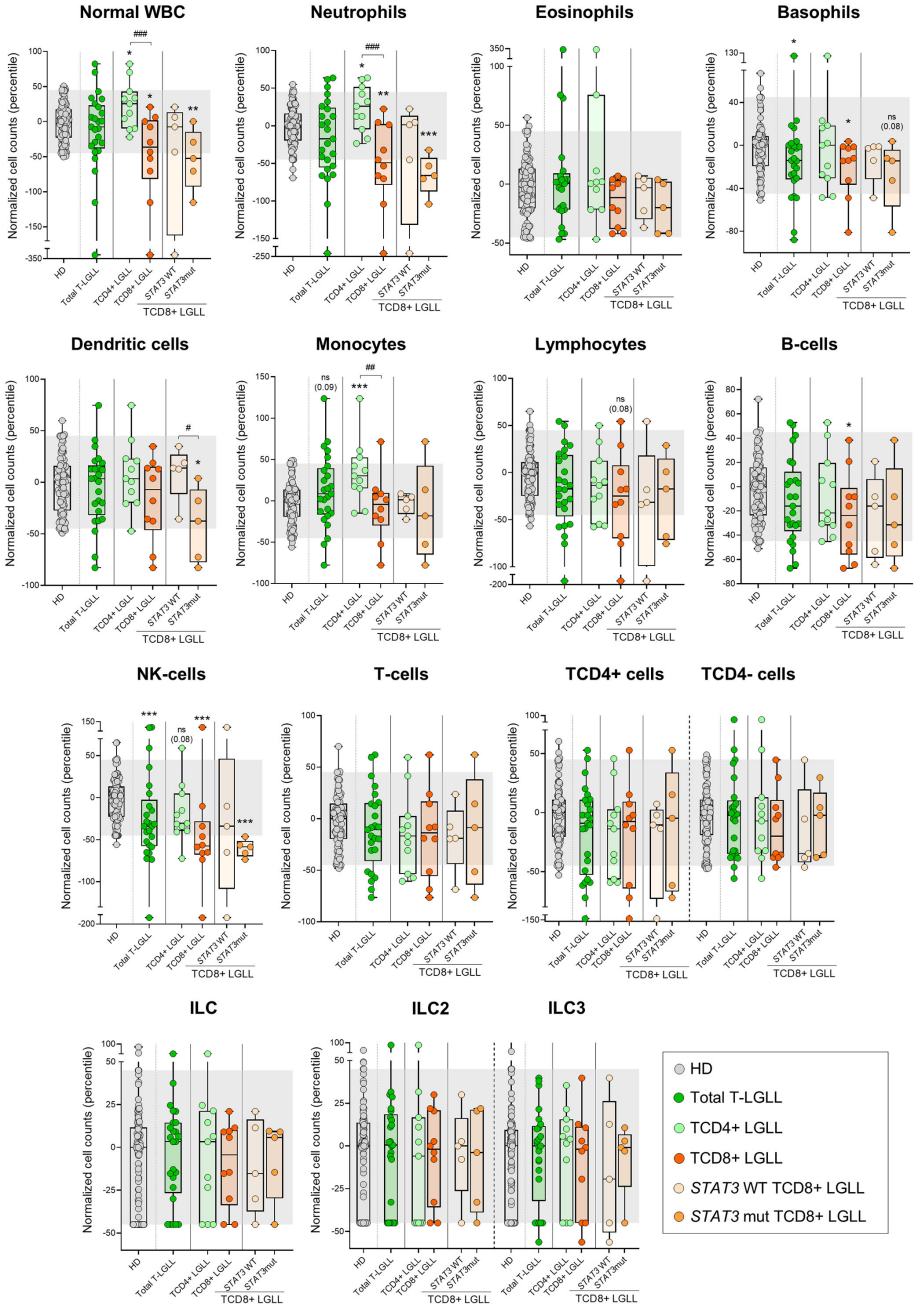
Distribution of the major normal/residual PB leukocyte subsets in blood of T-LGLL patients and their major T-LGLL TαβCD4+ and TαβCD8+ subtypes. Box plots show the distribution of normalized counts of WBC populations in blood (percentiles values relative to age-matched HD reference ranges) for T-LGLL patients. In all graphs, dots correspond to individual samples, while notched boxes represent the 25^th^ and 75^th^ percentile values; the lines inside the box correspond to median values (50^th^ percentile=0) and whiskers represent minimum and maximum values. Gray dots represent samples collected from HD. Dark green dots denote total T-LGLL cases, while light green and orange dots correspond to the TαβCD4+ and TαβCD8+ subtypes, respectively. Light orange and intermediate orange dots represent TCD8+ cases with *STAT3* wild-type and *STAT3*-mutated profiles, respectively. *p-value ≤0.05, **p-value ≤0.01 and ***p-value ≤0.001 *vs*. HD. Trends versus HD or between the TαβCD4+ LGLL and TαβCD8+ LGLL groups are shown for p-values <0.1. P-values resulting from comparisons between the two T-LGLL lineages or TαβCD8+ LGLL with different *STAT3* mutational status are depicted as ^#^p-value ≤0.05, ^##^p-value ≤0.01 and ^###^p-value ≤0.001. The gray shading corresponds to the 5^th^-95^th^ percentiles (values -45 and 45, respectively). (alphabetical order): HD, healthy donor; ILC, innate lymphoid cell; mut, mutated; NK, natural-killer; ns, statistically not significant; T-LGLL, T-large granular lymphocytic leukemia; WBC, white blood cell; WT, wild-type.

In contrast to both T-PLL and SS/MF, T-LGLL cases displayed overall normal counts in blood of all major leukocyte populations analyzed (**Figure 3**, **Supplementary Table 5**), except for significantly reduced (*vs*. HD) basophil (p=0.033) and NK-cell numbers (p=0.001). Despite this global behavior, when we separately considered the two major groups of TαβCD4+ and TαβCD8+ T-LGLL, several additional alterations (compared to HD) emerged. Thus, while the (normal) WBC and neutrophil counts were significantly increased (*vs*. HD) in TαβCD4+ LGLL (p=0.021 and p=0.012, respectively), they were abnormally low (*vs*. HD) in TαβCD8+ LGLL cases (p=0.013 and p=0.002, respectively), with statistically significant differences for these two cell populations between both T-LGLL subtypes (p=0.004 and p=0.002, respectively). In addition, total monocyte counts were also increased (*vs*. HD) in TαβCD4+ (but not TαβCD8+) T-LGLL cases (p=0.001), while significantly reduced numbers of basophils (p=0.036), B-cells (p=0.040), and particularly NK-cells (p<0.001), were exclusively found among TαβCD8+ T-LGLL patients (**Figure 3**, **Supplementary Table 5**). Of note, within TαβCD8+ LGLL cases, distinct profiles were further observed depending on the presence *vs*. absence of *STAT3* mutations, since the reduced (*vs*. HD) normal WBC (p=0.007), neutrophil (p<0.001), basophil (p=0.08), dendritic cell (p=0.028) and NK-cell (p<0.001) counts found among TαβCD8+ LGLL cases were exclusively observed among those patients that carried *STAT3* mutations, while normal (p>0.05) in *STAT3* wild-type cases (**Figure 3**, **Supplementary Table 5**). Likewise, decreased numbers of normal WBC (p<0.001), neutrophils (p<0.001), basophils (p=0.04), NK-cells (p<0.001) and B-cells (p=0.013) were more pronounced (*vs*. HD) among TαβCD8+ LGLL cases with a CR- *vs*. CD183^+^ phenotype (**Supplementary Figure 4**). In case of T-LGLL, no association with other clinical features was found (different from those associated to *STAT3* mutations in TαβCD8+ LGLL), and in all cases with follow-up data (n=17), the disease remained stable without therapy.

### Altered distribution in blood of maturation-associated and functionally-related T-cell subsets in T-PLL, SS/MF and T-LGLL patients

**Figure 4:**
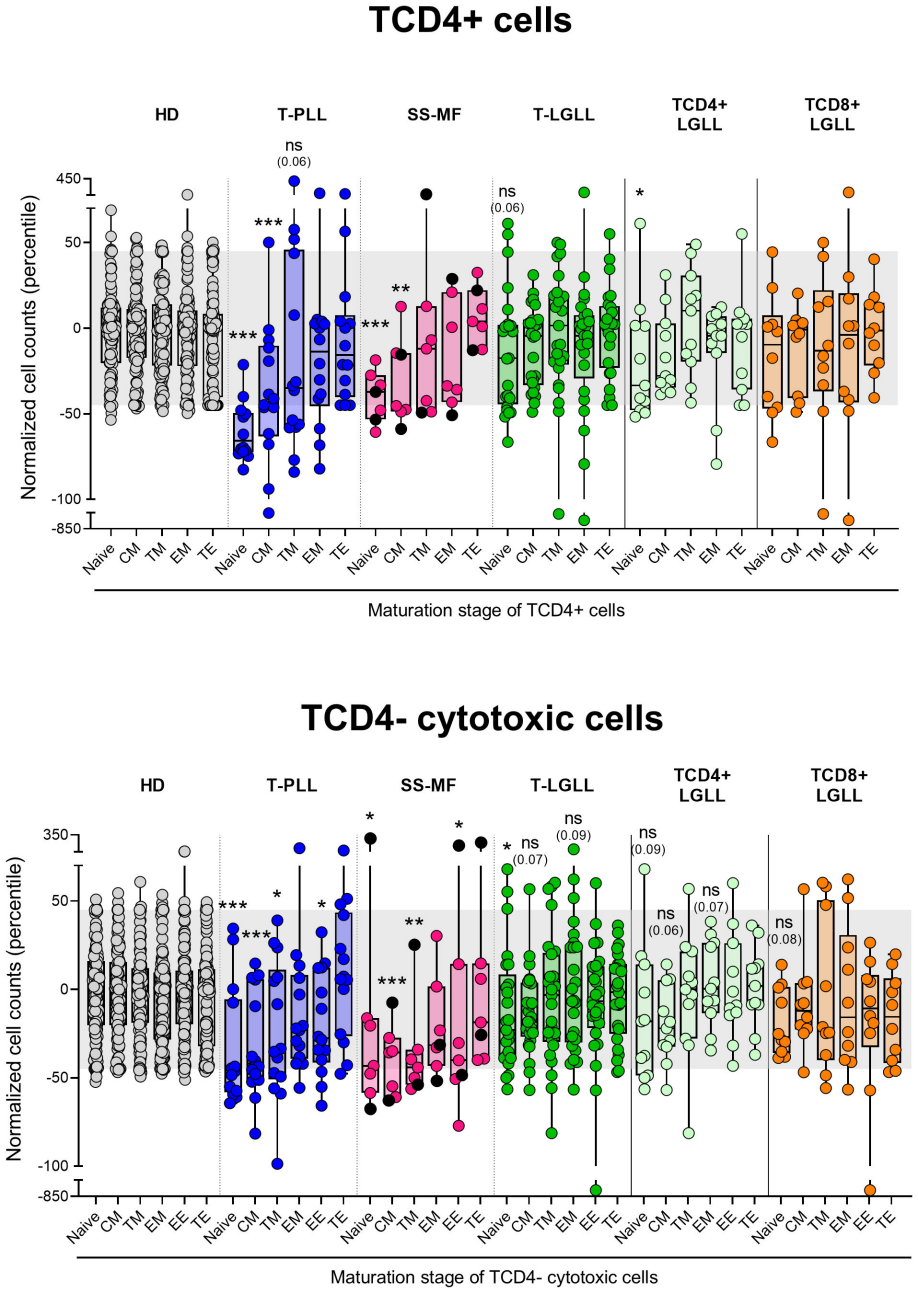
Distribution intro different T-cell maturation stages of normal/residual TCD4+ and TCD4- cells according to the diagnostic category of TCLPD. Box plots show the distribution of normalized blood counts of normal residual TCD4+ and TCD4- cells at different maturation stages (percentile values relative to age-matched HD reference ranges) for T-CLPD patients. In all graphs, dots correspond to individual samples, while notched boxes represent the 25^th^ and 75^th^ percentile values; the lines inside the box correspond to median values (50^th^ percentile=0) and whiskers represent minimum and maximum values. Gray dots represent HD cases; dark blue dots denote T-PLL cases; light pink and black dots represent SS and MF cases, respectively; dark green dots denote total T-LGLL cases, while light green and orange dots correspond to the TαβCD4+ and TαβCD8+ LGLL subtypes, respectively. *p-value ≤0.05, **p-value ≤0.01 and ***p-value ≤0.001 *vs*. HD. Trends versus HD are shown for p-values ≤0.1. The gray shading corresponds to the 5^th^-95^th^ percentiles (values -45 and 45, respectively). (alphabetical order): CM, central memory; HD, healthy donors; EE, early effector; EM, effector memory; MF, mycosis fungoides; ns, statistically not significant; SS, Sézary syndrome; TE, terminal effector; T-LGLL, T-large granular lymphocytic leukemia; TM, transitional memory; T-PLL, T-prolymphocytic leukemia.

Despite total TCD4+ cells were only decreased significantly (*vs*. HD) among T-PLL (p<0.001) and SS/MF (p=0.004) cases, the overall number of naive TCD4+ cells was reduced (p ≤ 0.06 *vs*. HD) in all three categories of mature T-cell neoplasms investigated here. However, such decrease was more pronounced in T-PLL (p<0.001), where it also involved CM (p<0.001) TCD4+ cells, followed by SS/MF who also showed lower naive (p=0.001) and CM (p=0.007) cell counts, while T-LGLL patients displayed milder/borderline decreased numbers of (only) TCD4+ naive cells (p=0.06 *vs*. HD), exclusively among TαβCD4+ (but not TαβCD8+) LGLL cases (**Figure 4**). Of note, an even more pronounced decrease of naive and CM cells (*vs*. HD) was observed in the three T-CLPD group subtypes within the TCD4- cytotoxic-cell compartment, where reduced TCD4- cell numbers extended beyond both subsets to more advanced maturation stages compared to the TCD4+ cell compartment (**Figure 4**). Thus, a significant reduction in naive (p ≤ 0.018), CM (p ≤ 0.002) and TM (p ≤ 0.043) TCD4- cytotoxic cells was observed in both T-PLL and SS/MF cases, whereas in T-LGLL only the naive (p=0.043) and CM (p=0.034) TCD4- cytotoxic cell compartments were abnormally reduced in blood (**Figure 4**).

**Figure 5:**
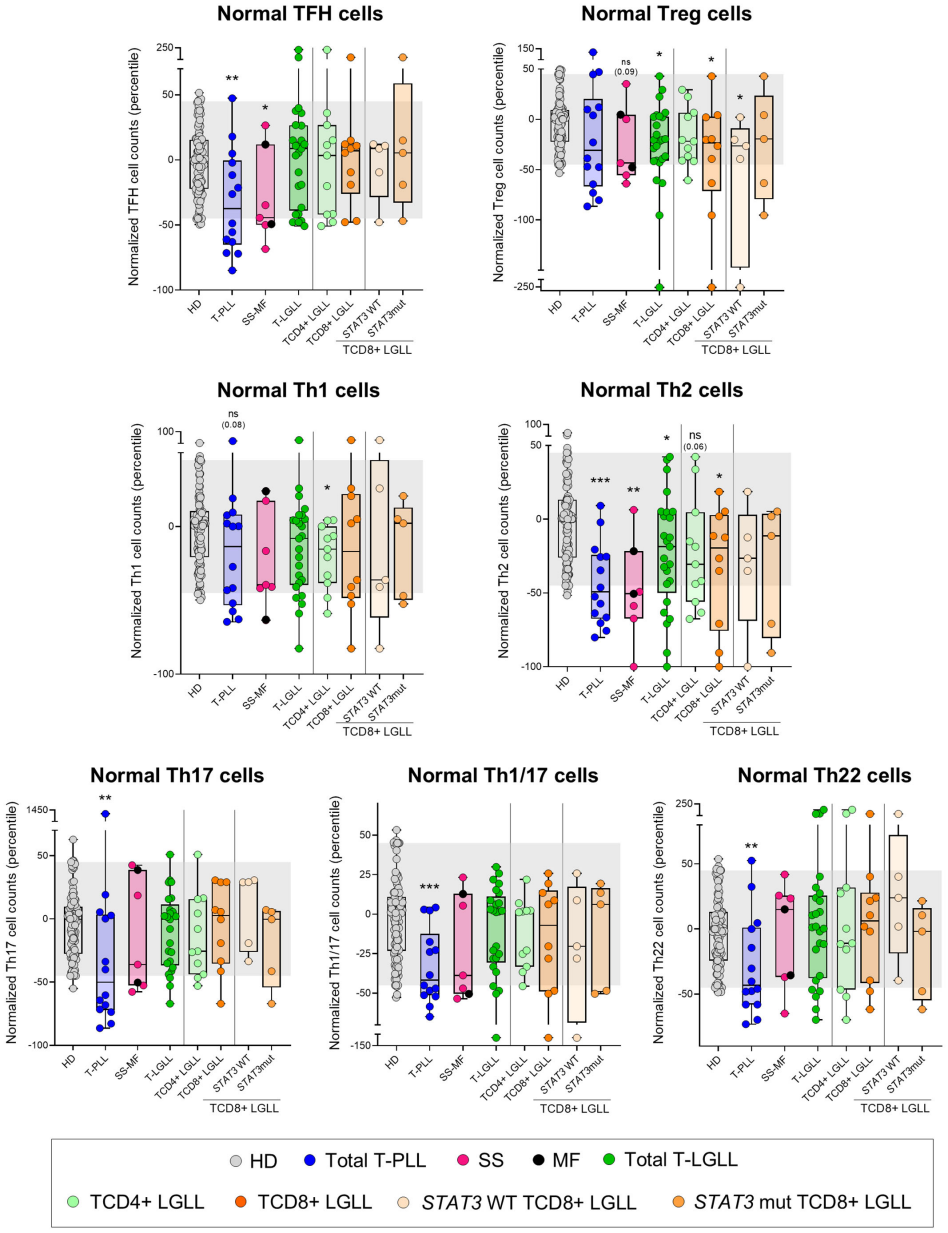
Distribution of major normal/residual TCD4+ subsets in blood according to the diagnostic category of T-CLPD. Box plots show the distribution of normalized blood counts of T-follicular helper (TFH) cells, T regulatory (Treg) cells, Th1, Th2, Th17, Th1/17 and Th22 cells in blood (percentile values relative to age-matched HD reference ranges) for T-CLPD patients. In all graphs, dots correspond to individual samples, while notched boxes represent the 25^th^ and 75^th^ percentile values; the lines inside the box correspond to median values (50^th^ percentile=0) and whiskers represent minimum and maximum values. Gray dots represent HD cases; dark blue dots denote T-PLL cases; light pink and dark pink dots represent SS and MF cases, respectively; dark green dots denote total T-LGLL cases, while light green and orange dots correspond to the TαβCD4+ and TαβCD8+ LGLL subtypes, respectively. Light orange and intermediate orange dots represent TαβCD8+ cases with *STAT3* wild-type and *STAT3*-mutated profiles, respectively. *p-value ≤0.05, **p-value ≤0.01 and ***p-value ≤0.001 *vs*. HD. Trends versus HD are shown for p-values ≤0.1. The gray shading corresponds to the 5^th^-95^th^ percentiles (values -45 and 45, respectively). (alphabetical order): HD, healthy donor; MF, mycosis fungoides; mut, mutated; ns, statistically not significant; SS, Sézary syndrome; T-LGLL, T-large granular lymphocytic leukemia; T-PLL, T-prolymphocytic leukemia; WT, wild-type.

In T-PLL, the above T-cell alterations led to a pronounced and extensive reduction in blood of all functional subsets of TCD4+, except Treg and Th1 cells, consisting of significantly decreased counts (*vs*. HD) of TFH (p=0.004), Th2 (p<0.001), Th17 (p=0.007), Th1/17 (p<0.001) and Th22 (p=0.004) cells (**Figure 5**, **Supplementary Tables 6**, **7**). Notably, TFH (p=0.022 and p=0.003), Th17 (p=0.001 and p=0.01) and Th22 (p=0.018 and p=0.012) cell populations were also more deeply reduced (*vs*. HD) in patients with a naive/naive-CM tumor cell phenotype and those whose tumor cells exhibited ≥2 profiles than in more mature and functionally more homogeneous T-PLL cases (**Supplementary Figure 5**); in contrast, no differences were found among the above T-PLL subgroups regarding response to treatment and disease progression. Likewise, SS/MF was characterized by a significant reduction (*vs*. HD) in TFH (p=0.024) -particularly in SS/MF cases with a Th17 phenotype of tumor cells-, and Th2 cells (p=0.002), associated with normal counts of all other functional subsets of TCD4+ cells investigated (**Figure 5**, **Supplementary Tables 8**, **9**, **Supplementary Figure 5**). In turn, T-LGLL cases presented decreased Tregs (p=0.014), particularly within the *STAT3* wild-type (p=0.038) and CR- (p=0.036) TαβCD8+ subset of T-LGLL, together with abnormally low Th2 (p=0.037) and Th1 cell numbers, TαβCD4+ LGLL cases (p=0.036) (**Figure 5**, **Supplementary Tables 10**, **11**, **Supplementary Figure 5**).

When dissecting the different functional Th-cell compartments according to their maturation stage, the changes observed in each Th subset resembled those described above for the whole TCD4+ cell compartment per T-CLPD category (**Supplementary Figure 6**). Specifically, in T-PLL reduced counts across most maturation stages for all Th subpopulations (p ≤ 0.045) were observed, whereas in SS/MF decreased counts of CM (p<0.001) and TM (p=0.024) Th1 cells, and both CM and EM Th2 cells (p=0.007 and p<0.001, respectively) were found. In T-LGLL a reduction in Th1 and Th2 CM cells (p=0.01 and p=0.04) together with an increase in Th1/17 TE cells (p<0.001) were the main findings (**Supplementary Figure 6**).

## Discussion

Dissection of those alterations involving the tumor microenvironment across various types of cancer, including hematological malignancies, together with a better understanding of the underlying pathogenic mechanisms, have become increasingly important, due to the progressively greater benefits of an increasingly larger number of novel targeted immunotherapies (9, 10). However, at present, there is limited knowledge about the potentially altered tumor-associated immune microenvironment in mature T-cell neoplasms (19, 24, 36). Here, we investigated in detail the potentially altered immune cell profiles present in the blood of (leukemic) T-CLPD, by taking T-PLL, SS/MF and T-LGLL as models of this group of diseases. Our results demonstrated for the first time that these mature T-cell neoplasms exhibit pronounced alterations across different immune cell compartments, that encompass both the innate and adaptive immunity. Among the three T-CLPD diagnostic categories investigated, T-PLL (which is characterized by a leukemic expansion of mostly naive/CM post-thymic tumor T-cells (15, 17)), displayed the most prominently altered immune cell profiles, compared to SS/MF, and particularly to T-LGLL, in which the leukemia/lymphoma cells display a predominant CM/TM and TE phenotype, respectively (18, 19, 23–25).

Previous reports have recurrently shown that pronounced leukocytosis and lymphocytosis, due to the expansion and dissemination of leukemia cells in blood, are a hallmark of T-PLL (29). Here, we further demonstrated that the increased WBC count is not solely attributable to the excessive number of circulating T-PLL cells (though they are the major contributor to leukocytosis, as they massively infiltrate the blood), but also to a parallel abnormal elevation of other (normal) residual WBC populations. Specifically, antigen presenting cells (APC), such as dendritic cells and monocytes, and several lymphoid cell subsets other than (normal) T-cells, such as B-cells, NK-cells and ILC (mostly at the expense of ILC3 cells and, to a lesser extent also, ILC2 cells in TCD4+ PLL) were increased, particularly (APC and ILC2) in patients who showed disease progression *vs*. the non-progressors. In contrast, normal T-cells were severely reduced, at the expense of both TCD4+ and TCD4-cytotoxic cell compartments. The increased APC and NK-cell counts observed in blood of T-PLL patients were somehow unexpected in a highly aggressive disease such as T-PLL (particularly if we consider also the even higher monocyte blood counts in T-PLL patients who showed disease progression *vs*. stable disease), since higher numbers of these immune cell populations have been recurrently associated with stronger anti-tumor responses in several cancer types (38–40). However, it has also been demonstrated that both dendritic cells and NK-cells from patients with other types of leukemias (e.g., acute myeloid leukemia and chronic myeloid leukemia) and lymphomas, display phenotypic and functional features compatible with an impaired anti-tumor functionality (41, 42). Taken together, these findings point out the need for a more in-depth phenotypic and functional characterization of blood monocytes, dendritic cells and NK-cells in T-PLL, to determine the pathogenic relevance of our observations.

In contrast to dendritic cells and NK-cells, whose role in controlling tumor growth is relatively well-established in several cancer models (39, 40, 43), the involvement of ILC in shaping the tumor microenvironment remains poorly understood and controversial, since ILC have been implicated in both anti-tumoral and pro-tumoral responses (44, 45). As an example, ILC3 have been suggested to contribute to the generation and maintenance of tertiary lymphoid structures (44, 45) (which have been associated with a better prognosis) and to exhibit cytotoxic activity against melanoma cells (44–46); likewise, in murine models, tumor-infiltrating ILC3 have been shown to transdifferentiate into anti-tumor ILC1-like cells (44). In contrast, ILC3 have also been found to promote tumor progression via secretion of high levels of IL-17 and IL-22 (47–49), which in turn have been related in solid tumors with metastatic progression, tumor growth (47, 48), and chemoresistance (49), respectively. Like ILC3, ILC2 have also been associated with impaired anti-tumor responses and a poor prognosis in many cancer types (44). The greater ILC2 numbers found in T-PLL patients who progressed *vs*. those who showed stable disease would further support previous observations about the association of this ILC subset with worse cancer prognosis (44). At present, it is well-established that these effects are mainly mediated by an increased recruitment of dendritic cells and/or enhancement of their anti-tumor activity through their dependence on IL-33, which might also contribute to explain the here reported increased counts in blood of circulating monocytes and NK-cells, in addition to ILC2, since these cell populations share in common the expression of the ST2 IL-33 receptor (50).

Despite a (milder) increase in the normal WBC count was also observed in SS/MF, in contrast to T-PLL, this increase was not due to greater APC and lymphoid cells other than normal T-cells. Conversely, the elevation of normal WBC levels in SS/MF was primarily due to increased neutrophil counts, consistent with earlier observations (19, 51). In contrast, dendritic cells, total lymphocytes, NK-cells and total T-cells and their major TCD4+ cells and TCD4- cytotoxic cell compartments, were all reduced in blood compared to age-matched HD. Previous studies have also reported a decrease in circulating NK-cells and TCD8+ cells in SS/MF (19, 52), along with an increased infiltration of the skin by TCD8+ cells, NK-cells and dendritic cells (53, 54). These findings point to an increased migration of T/NK cytotoxic cells and dendritic cells from PB to the (lesional) skin of SS/MF patients. However, the specific role of tissue-infiltrating dendritic cells, TCD8+ cytotoxic cells and NK-cells remains to be defined, since conflicting data exists regarding whether they (locally) promote or inhibit tumor growth (52, 53). An additional potential limitation of our study relies on the relatively small cohort of SS/MF patients investigated, and the more limited statistical power associated with our findings.

As previously reported, T-LGLL patients behaved differently, depending on the TCD4+ *vs*. TCD8+ nature of clonal T-cells and the presence *vs*. absence of *STAT3* mutations (25, 55). Thus, (mildly) increased numbers of normal WBC, primarily due to an elevation of both the neutrophil and monocyte counts, were restricted to TαβCD4+ LGLL patients. Conversely, TαβCD8+ LGLL (and specifically TαβCD8+ LGLL with *STAT3* mutations) was typically associated with reduced normal WBC and neutrophil counts. In addition, basophils and NK-cells, and to a lesser extent also B-cells, were all abnormally reduced in blood of TαβCD8+ cases, in contrast to both TCD4+ LGLL and *STAT3* wild-type TαβCD8+ LGLL, where normal levels of these cell populations were found. These results confirm in a new cohort of patients that the altered immune cell profiles observed in patients with TαβCD8+ LGLL are primarily driven by *STAT3* mutations (36); in addition, here we also show that these immune cell alterations go beyond the recurrently reported neutropenia (55, 56), since multiple other WBC compartments were affected in our cohort such as NK-cells, dendritic cells and, to a lesser extent also, basophils, in addition to the neutrophils. Altogether, these results suggest that the presence of decreased numbers in blood of the above referred leukocyte subsets in TαβCD8+ LGLL might be a surrogate marker of an underlying *STAT3* mutation in these patients. It should be noted that *STAT3* mutations (present in 40% to 50% of T-LGLL) are the most common type of gain-of-function (GOF) genetic lesions among T-LGLL patients. Presence of *STAT3* mutations in T-LGLL is associated with a worse prognosis (*vs*. *STAT3* wild-type cases), due to a higher frequency of cytopenias (i.e., neutropenia, anemia and to a lesser extent also thrombocytopenia), and other diseases related to dysregulation of T-cell–mediated immunity, such as acquired pure red cell aplasia and rheumatoid arthritis, among other autoimmune and inflammatory diseases, and a higher prevalence of (second) tumors (56). Of note, genetic abnormalities, particularly *STAT3* GOF mutations, have been regarded as a potential molecular mechanism involved in the development of autoimmunity (i.e., cytotoxic T-cell clones have been hypothesized to perpetuate tissue damage both directly and by inducing autoantigens) (56). Thus, early identification of decreased blood counts of leukocyte subset other than neutrophils (observed exclusively among *STAT3* mutated cases) might be of relevance for the clinical management of TαβCD8+ LGLL patients.

More in-depth analysis of the (normal) blood circulating T-cell compartment in T-PLL and SS/MF revealed similarly altered profiles, which consisted of a marked reduction in the more immature (naive and CM) compartments of both T-helper (Th) and T-cytotoxic cells, with relatively lower involvement of more mature (memory and effector) T-cells. In contrast, in T-LGLL, T-cell counts in blood were within the normal reference ranges, except for a minor reduction of the naive/CM T-cell compartment. These findings point to a defective T-cell production (including both the TCD4+ and TCD8+ major populations) in T-PLL and SS/MF, and to a much lesser extent also in T-LGLL. Despite the mechanisms responsible for the decreased T-cell production remain currently unknown, a shift in the (thymic) production of normal-to-tumor (e.g., naive) T-cells, at least in T-PLL, where naive tumor T-cells predominate in parallel to a severe normal naive T-cell defect, associated or not with thymic infiltration by tumor cells, might contribute to explain our findings. Alternatively, the secretion of cytokines by tumor cells (that have been shown to act as Th cells with various functional-associated profiles (17, 19, 24, 25, 57) might directly or indirectly inhibit T-cell production in the thymus (58). Upon considering the distribution of the different TCD4+ functional cell subsets in blood, we found TFH cells to be significantly reduced in both T-PLL and SS/MF, but not in T-LGLL. These findings confirm and extend on previous observations specifically reported in SS (19), extended now also to T-PLL patients. Of note, TFH cells have been associated with better outcomes, primarily in solid tumors (59), suggesting that their reduction in T-PLL and SS/MF patients may also confer a poorer prognosis. Likewise, Treg cell counts were decreased in (*STAT3* wild-type) TαβCD8+ LGLL. Tregs are known to induce significant immunosuppressive activity, and to play a critical role in tumor cell growth, proliferation and survival in patients with hematological malignancies other than T-CLPD (60). If this holds true, the lower Treg cell counts here reported among *STAT3* wild-type TCD8+ LGLL, might contribute to explaining the better prognosis of this disease group compared to T-PLL, SS/MF and also *STAT3*-mutated TαβCD8+ LGLL. In parallel, a significant reduction of most subsets of Th-cells was observed in T-PLL, highlighting the profound immunodeficiency associated with this neoplasm. In turn, Th2 cells were the only Th subset severely decreased in SS/MF, while (only) mildly reduced in T-LGLL. Since both Th2 and Treg cells express high levels of CD194 (30, 61), a therapeutic target in SS/MF patients (62), such treatments could further deplete these subpopulations (63), and therefore, potentially exacerbate the pre-established immunodeficiency status in these patients. Since these immune profiles were identified at the time of T-CLPD diagnosis, analyzing in parallel their evolution with the disease outcome through longitudinal studies including larger cohorts of patients would be highly valuable to know in a comprehensive way how the immune changes contribute to clinical manifestations and patient outcomes.

In summary, our findings reveal that all three T-CLPD disease subtypes here analyzed display (variable) alterations in blood across all immune cell compartments, encompassing both innate and adaptive immunity. The degree of impairment of the immune system highly depends on the diagnostic category of the disease, with a severe to moderate and milder immunodeficiency, paralleled by a progressively lower alteration of the earliest maturation stages of normal residual T-lymphocytes in T-PLL, SS/MF and T-LGLL, respectively. The pathogenic mechanisms leading to the altered immune cell profiles, as well as their clinical implications, deserve further investigations in larger cohorts of T-CLPD patients, extended also to other diagnostic subtypes of the disease.

## Data Availability

The raw data supporting the conclusions of this article will be made available by the authors, without undue reservation.
