# Supplementary material for: Altered immune cell profiles in blood of mature/peripheral T-cell leukemia/lymphoma patients: an EuroFlow study
Source: Front Immunol. 2025 Mar 21;16:1561152. doi: 10.3389/fimmu.2025.1561152 (PMC11968749; doi:10.3389/fimmu.2025.1561152)
Supplement: Supplementary file 1 [file DataSheet1.docx]

**SUPPLEMENTARY MATERIAL**

**TITLE: *Altered immune cell profiles in blood of mature/peripheral T-cell leukemia/lymphoma patients: an EuroFlow study***

**SUPPLEMENTARY METHODS**

***Classification of normal TCD4+ cells into T helper (Th), T-regulatory (Tregs) and follicular T-helper (TFH) cells, and their maturation-associated and functional Th-related subpopulations.*** In a first step, the neoplastic cell population was identified in every patient sample, based on the presence of leukemia/lymphoma-associated immunophenotypes (LAIPs) and/or a monomodal TRBC1 expression profile. Then, this tumor cell population was excluded for further immunophenotypic analyses. Subsequently, the remaining normal leukocytes present in blood of each individual patient, as well as all leukocytes from healthy donors (HD) blood, were classified into the different major leukocyte populations. Afterwards, in-depth dissection of TCD4+ cells into their different maturation-associated and functional-related phenotypic subsets was performed, following gating strategies which have been previously described in detail (1,2). Briefly, in a first step TCD4+ cells were categorized as conventional T helper (Th) (non-TFH and non-Treg) cells, regulatory T cells (Tregs; CD3^+^CD4^+^CD25^++^CD127^-/lo+^), or follicular T-helper cells (TFH; CD3^+^CD4^+^CD185^+^) cells, based on their differential expression of CD25, CD127 and CD185 (1,2). Next, the classical Th1, Th2, Th17, Th1/17 and Th22 subpopulations, as well as non-classical Th subsets were identified, according to the pattern of expression of the CD183, CD194, CD196 and CCR10 chemokine receptors (1,2): Th1 cells were identified as CD183^-/lo/+^ CD194^-^ CD196^-^ CCR10^-^ cells, Th2 as CD183^-^ CD194^+^ CD196^-^ CCR10^-^, Th17 as CD183^-^ CD194^+^ CD196^+^ CCR10^-^ TCD4+ cells, Th1/17 as CD183^+^ CD194^-^ CD196^+^ CCR10^-^ cells and Th22 cells were defined as CD183^-^ CD194^+^ CD196^+^ CCR10^+^ TCD4+ cells; in addition the other cell populations were also identified based on the following phenotype: CD183^+^ CD194^+^ CD196^+^ CCR10^-/+^, CD183^+^ CD194^+^ CD196^-^ CCR10^-/+^, CD183^-^ CD194^-^ CD196^+^ CCR10^-^, CD183^-^ CD194^+^ CD196^-^ CCR10^+^, CD183^+^ CD194^-^ CD196^-/+^ CCR10^+^ and CD183^-^ CD194^-^ CD196^-^ CCR10^-^ non-conventional Th cell subsets (1,2). In parallel, the CD27, CD45RA and CD62L maturation-associated markers were used to identify naive cells (CD45RA^hi^ CD27^hi^ CD62L^hi^ cells characterized by the lack of expression of the above chemokine receptors), central memory (CM; CD45RA^-^ CD27^+^ CD62L^+^), transitional memory (TM; CD45RA^-^ CD27^+^ CD62L^-^), effector memory (EM; CD45RA^lo/+^ CD27^-^ CD62L^-/+^), early effector (EE; CD45RA^+^ CD27^+/lo^ CD62L^-/+^) (only among CD4^-^ T cells), and terminal effector (TE; CD45RA^+^ CD27^-^ CD62L^-/+^) cells, within both total TCD4+ and TCD4- cytotoxic cells, as well as within each of the functional Th-subsets of TCD4+ cells (1).

***Assessment of T-cell clonality and molecular/genetic characterization of T-LGLL and T-PLL patients*** The clonal nature of the aberrant/suspicious tumor T-cell population present in each patient was confirmed by either flow cytometry -through analysis of the T-cell receptor Vβ (TCRVβ) repertoire (using the IOTest® Beta Mark TCR-Vβ Repertoire Kit (Beckman-Coulter, Brea, CA) and/or based on monotypic expression of TRBC1 (3)- and/or by the presence of single (or a few dominant) TCRβ and/or TCRγ V(D)J gene rearrangements on highly-purified (phenotypically aberrant) T-cells (purity ≥95%; FACSAria-III flow cytometer, BD), based on the BIOMED-2 probes and polymerase chain reaction (PCR) assays (4). Moreover, *STAT3* and *STAT5b* gene mutations were investigated on purified (FACS-sorted) clonal T-cells from T-LGLL patients, following previously described methods (5). Fluorescence in situ hybridization (FISH) studies were performed to screen for the presence of genetic alterations involving the *TCL1* (T-cell leukemia/lymphoma1) family of genes on FACS-purified T-PLL cells (6–8).

**SUPPLEMENTARY TABLES**

**Supplementary Table 1.** Combinations of fluorochrome-conjugated antibody reagents used for the classification of patients with T-cell malignancies into specific WHO-2022 diagnostic categories.

1. **EuroFlow Lymphocyte Screening Tube (LST) ***

| **LST** | | | | | | | | |
| --- | --- | --- | --- | --- | --- | --- | --- | --- |
| **Tube** | **PacB** | **PacO** | **FITC** | **PE** | **PerCPCy5.5** | **PECy7** | **APC** | **APCH7** |
| 1 | CD4 + CD20 | CD45 | CD8 + Anti-λ | CD56 + Anti-κ | CD5 | CD19 + TCRγδ | CD3 | CD38 |

1. **EuroFlow T-cell CLPD panel ***

| **T-cell CLPD panel** | | | | | | | | |
| --- | --- | --- | --- | --- | --- | --- | --- | --- |
| **Tube** | **PacB** | **PacO** | **FITC** | **PE** | **PerCPCy5.5** | **PECy7** | **APC** | **APCH7** |
| 1 | CD4 | CD45 | CD7 | CD26 | SmCD3 | CD2 | CD28 | CD8 |
| 2 | CD4 | CD45 | CD27 | CD197 | SmCD3 | CD45RO | CD45RA | CD8 |
| 3 | CD4 | CD45 | CD5 | CD25 | SmCD3 | HLADR | CyTCL1 | CD8 |
| 4 | CD4 | CD45 | CD57 | CD30 | SmCD3 | - | CD11c | CD8 |
| 5 | CD4 | CD45 | CyPER | CyGRA | SmCD3 | CD16 | CD94 | CD8 |
| 6 | CD4 | CD45 | - | CD279 | SmCD3 | - | - | CD8 |

^*^ Ref. (9) in Supplementary bibliography.

For the two panels, the “stain & lyse” EuroFlow SOP were used, including a protocol for staining of cell surface membrane markers only for LST ([**www.EuroFlow.org**](http://www.EuroFlow.org)), and in combination with an SOP for combined staining for cell surface membrane antigens and intracellular molecules based on the Fix & Perm^TM^ reagent kit (Thermo Fisher Scientific, Waltham, MA) used according to the recommendations of the manufacturer and the EuroFlow SOP ([**www.EuroFlow.org**](http://www.EuroFlow.org)) for the T-cell CLPD panel.

Abbreviations (alphabetical order): APC, allophycocyanine; APCH7, allophycocyanine-Hilite^®^7; CLPD, chronic lymphoproliferative disorder; Cy, cytoplasmic; FITC, fluorescein isothiocyanate; GRA, granzyme B; PacB, Pacific Blue™; PacO, Pacific Orange™; PE, phycoerythrin; PECy7, phycoerythrin-cyanine 7; PerCPCy5.5, peridinin–chlorophyll protein-cyanine 5.5; PER, perforin; Sm, surface membrane; SOP, standard operating procedure.

**Supplementary Table 2.** Combination of fluorochrome-conjugated monoclonal antibody clones and their source used for the in-depth analysis of normal residual T-cells from T-CLPD patients.

|  | | | | |
| --- | --- | --- | --- | --- |
| Panel | **mAb** | **Fluorochrome** | **Clone** | **Source** |
| *Modified TCD4+ EuroFlow IMM Tube*^*^ | **CD2** | PacB | TS1/8 | BioLegend |
|  | **CD3** | BV786 | SK7 | BD |
|  | **CD4** | APCH7 | SK3 | BD |
|  | **CD7** | BUV661 | M-T701 | BD |
|  | **CD8** | BUV395 | RPA-T8 | BD |
|  | **CD25** | VioBright FITC | 4E3 | Miltenyi |
|  | **CD27** | BV421 | MT271 | BD |
|  | **CD45** | AF700 | HI30 | BD |
|  | **CD45RA** | BV510 | HI100 | BD |
|  | **CD62L** | BV650 | DREG56 | BioLegend |
|  | **CD127** | BV711 | HIL7RM21 | BD |
|  | **CD183** | PE | 1C6/CXCR3 | BD |
|  | **CD185** | APC | REA103 | Miltenyi |
|  | **CD194** | PE Cy7 | L291H4 | BioLegend |
|  | **CD196** | PE CF594 | 11A9 | BD |
|  | **Anti-CCR10** | PerCP Cy5.5 | 1B5 | BD |
|  | **Anti-TRBC1** | BUV737 | JOVI.1 | BD |

^*^ Ref. (1) in Supplementary bibliography.

Patient samples and normal blood samples from healthy donors (HD) were stained with the same combination of antibodies shown here, but without CD2, CD7, CD8 and anti-TRBC1. In all patient and HD samples, the “stain & lyse” EuroFlow SOPs for staining of cell surface membrane markers only, available at **www.EuroFlow.org**, was used.

Abbreviations (alphabetical order): AF, AlexaFluor; APC, allophycocyanine; APCH7, allophycocyanine–Hilite^®^ 7; BD, Becton Dickinson Biosciences; BUV, Brilliant Ultraviolet; BV, Brilliant Violet; CLPD, chronic lymphoproliferative disorder; FITC, fluorescein isothiocyanate; IMM, immune monitoring; mAb, monoclonal antibody; PacB, Pacific Blue™; PerCPCy5.5, peridinin–chlorophyll protein-cyanine 5.5; PE, phycoerythrin; PE-Cy7, PE-cyanine 7; SOP, standard operating procedure.

**Supplementary Table 3 |** Distribution of major normal/residual PB leukocyte populations in T-PLL and their TCD4+, TCD8+ and TCD4+CD8+ subgroups.

|  | **HD**  **(n=37)** | **Total T-PLL**  **(n=14)** | **T-PLL subtypes** | | | **P-value** |
| --- | --- | --- | --- | --- | --- | --- |
|  |  |  | **TCD4+ PLL (n=8)** | **TCD8+ PLL (n=2)** | **DP T-PLL**  **(n=4)** |  |
| **Total WBC** | 6230 (4010-11 340) | 36 350 (6500-209 000) | 48 450 (13400-209 000) | 83 450 (29 700-137 200) | 10 055 (6500-21 600) | ≤0.05^a,b,c,d^ |
| **Normal WBC** | 6230 (4010-11 340) | 11 196 (2769-36 933) | 14 625 (2769-36 933) | 13 452 (12 712-14 192) | 5499 (3573-7644) | ≤0.05^a,b,d^ |
| **Neutrophils** | 3426 (1190-7485) | 4492 (1344-28 201) | 6369 (1555-28 201) | 4325 (4068-4581) | 3048 (1344-4758) | NS (0.08^b^) |
| **Eosinophils** | 111 (0.30-571) | 129 (0-1718) | 269 (0-1718) | 6.0 (0-12) | 129 (59-482) | NS |
| **Basophils** | 48 (19-115) | 53 (22-181) | 53 (22-181) | 113 (46-180) | 76 (44-125) | ≤0.05^a^ |
| **Dendritic cells** | 7.1 (1.9-22) | 20 (0-229) | 40 (9.1-229) | 0 (0-0) | 18 (4.2-49) | ≤0.05^a,b^ |
| **Monocytes** | 434 (254-983) | 1497 (479-8698) | 2058 (479-8698) | 4003 (3665-4340) | 596 (572-742) | ≤0.05^a,b,d^ (0.08^c^) |
| **Lymphocytes** | 2083 (969-4556) | 2271 (626-5196) | 2420 (626-4950) | 3789 (2381-5196) | 1571 (1004-2160) | NS |
| **T-cells** | 1489 (618-2925) | 1004 (100-3621) | 1271 (100-3621) | 1686 (442-2930) | 697 (358-1132) | ≤0.01^c^ (0.07^a^) |
| **TCD4+ cells** | 957 (451-2102) | 387 (0-2930) | 382 (0-1173) | 1633 (336-2930) | 337 (65-650) | ≤0.01^a,b,c^ |
| **TCD4- cells** | 541 (142-1010) | 452 (0-2448) | 847 (100-2448) | 53 (0-106) | 430 (123-511) | NS |
| **NK-cells** | 294 (101-1344) | 580 (135-1366) | 463 (135-1366) | 1115 (892-1337) | 486 (377-609) | ≤0.05^a^ |
| **B-cells** | 228 (78-669) | 382 (196-2559) | 357 (204-1547) | 1951 (1342-2559) | 299 (196-656) | ≤0.05^a,b^ |
| **ILC** | 1.1 (0-8.1) | 5.0 (2.2-31) | 6.1 (3.1-20) | 17 (2.3-31) | 3.0 (2.2-5.5) | ≤0.05^a,b,c,d^ |
| **ILC2** | 0.48 (0-1.4) | 1.2 (0-7.0) | 1.4 (0-3.9) | 3.5 (0-7.0) | 0 (0-1.2) | ≤0.05^a,b,d^ |
| **ILC3** | 0.65 (0-7.3) | 4.4 (2.0-24) | 4.8 (2.0-16) | 13 (2.3-24) | 3.0 (2.2-4.3) | ≤0.01^a,b,c^ |
| **Tumor T-cells** | NA | 24 934 (2042-184 253) | 37 153 (3720-184 253) | 84 998 (46988-123 008) | 4999 (2042-13 956) | ≤0.05^d^ |

Results expressed as median (range) number of cells/ μl.

^a^ Total T-PLL *vs.* HD; ^b^ TCD4+ PLL *vs.* HD, ^c^ DP T-PLL *vs.* HD, ^d^ DP T-PLL *vs.* TCD4+ PLL.

Abbreviations (alphabetical order): DP, double (CD4+CD8+) positive; HD, healthy donor; ILC, innate lymphoid cell; NA, not applicable; NS, difference not statistically significant; PB, peripheral blood; T-PLL, T-prolymphocytic leukemia.

**Supplementary Table 4 |** Distribution of normal/residual PB leukocyte populations in blood of SS/MF patients.

|  | **HD**  **(n=21)** | **SS/MF**  **(n=7)** | **P-value** |
| --- | --- | --- | --- |
|  |  |  |  |
| **Total WBC** | 6390 (4410-11 300) | 13 600 (8200-20 800) | ≤0.001 |
| **Normal WBC** | 6390 (4410-11 300) | 8017 (4098-15 785) | NS (0.06) |
| **Neutrophils** | 3335 (1935-6978) | 5685 (3165-14 292) | ≤0.01 |
| **Eosinophils** | 108 (23-327) | 89 (14-401) | NS |
| **Basophils** | 42 (15-111) | 55 (2.4-278) | NS |
| **Dendritic cells** | 7.1 (0.85-19) | 1.7 (0.40-5.6) | ≤0.01 |
| **Monocytes** | 489 (222-958) | 568 (288-1597) | NS |
| **Lymphocytes** | 2157 (919-4128) | 1225 (555-4400) | NS (0.07) |
| **T-cells** | 1638 (551-3415) | 1012 (261-3955) | NS (0.095) |
| **TCD4+ cells** | 955 (330-2628) | 633 (158-1122) | NS (0.08) |
| **TCD4- cells** | 615 (167-1297) | 195 (103-2833) | NS (0.09) |
| **NK-cells** | 308 (78-1103) | 72 (12-381) | ≤0.001 |
| **B-cells** | 153 (29-345) | 158 (37-432) | NS |
| **ILC** | 0.85 (0-4.2) | 0.79 (0.44-2.1) | NS |
| **ILC2** | 0.39 (0.00-2.5) | 0.26 (0.00-0.81) | NS |
| **ILC3** | 0.46 (0.00-1.7) | 0.53 (0.18-1.5) | NS |
| **Tumor T-cells** | NA | 2895 (226-8283) | NA |

Results expressed as median (range) number of cells/ μl.

Abbreviations (alphabetical order): HD, healthy donor; ILC, innate lymphoid cell; MF, mycosis fungoides; NA, not applicable; NS, differences not statistically significant; SS, Sezary syndrome; PB, peripheral blood.

|  | **HD**  **(n=72)** | **Total T-LGLL**  **(n=26)** |  | **T-LGLL subtypes** | **P-value** |  |  |  |  |
| --- | --- | --- | --- | --- | --- | --- | --- | --- | --- |
|  |  |  | **TαβCD4+ (n=12)** | **TαβCD8+ (n=10)** | **TαβCD8+ *STAT3* WT**  **(n=5)** | **TαβCD8+ *STAT3* mutated**  **(n=5)** | **TαβDP**  **(n=2)** | **Tγδ**  **(n=2)** |  |
| **Total WBC** | 7160 (3840-12 090) | 7987 (1150-15 230) | 11875 (4566-15 230) | 7010 (1150-12 000) | 6840 (4200-9000) | 8114 (1150-12 000) | 5900 (4800-7000) | 4858 (4816-4900) | ≤0.01^b,d^ |
| **Normal WBC** | 7160 (3840-12 090) | 6291 (753-13 497) | 7815 (3800-13 497) | 5534 (753-8267) | 6562 (3988-8267) | 3890 (753-6534) | 5819 (4783-6855) | 4618 (4373-4864) | ≤0.05^c,d,e,g^ |
| **Neutrophils** | 3808 (1190-7536) | 3241 (49-7591) | 4524 (2212-7591) | 2218 (49-4908) | 3863 (1736-4908) | 1508 (49-2377) | 1775 (1330-2220) | 1902 (1583-2220) | ≤0.05^c,d,e,g^ |
| **Eosinophils** | 140 (0.93-613) | 120 (0-3053) | 120 (0-3053) | 99 (10-195) | 152 (54-195) | 87 (10-142) | 168 (132-203) | 387 (65-708) | NS |
| **Basophils** | 52 (9.5-179) | 37 (4.7-203) | 45 (12-203) | 36 (6.1-54) | 36 (26-54) | 36 (6.1-46) | 19 (4.7-33) | 37 (31-44) | ≤0.05^a,c,g^ |
| **Dendritic cells** | 8.7 (1.7-22) | 8.6 (0-25) | 8.6 (1.8-25) | 6.8 (0-17) | 11 (4.0-17) | 3.2 (0-8.8) | 6.9 (4.4-9.3) | 6.5 (2.5-11) | ≤0.05^e,g^ |
| **Monocytes** | 476 (251-1038) | 549 (149-1634) | 754 (268-1634) | 447 (149-921) | 493 (399-549) | 368 (149-921) | 573 (331-814) | 353 (244-462) | ≤0.05^b,d^ |
| **Lymphocytes** | 2238 (819-4609) | 1717 (506-4540) | 1699 (934-4540) | 1598 (506-3705) | 1524 (1474-3705) | 1672 (506-3363) | 3279 (2953-3604) | 1933 (1154-2712) | ≤0.05^a,c^ (0.06^b^) |
| **T-cells** | 1670 (525-3778) | 1293 (441-3451) | 1191 (670-3451) | 1291 (441-2927) | 1245 (1098-2193) | 1434 (441-2927) | 2479 (2443-2515) | 1434 (987-1881) | ≤0.05^b^ (0.07^a^) |
| **TCD4+ cells** | 1028 (324-2622) | 901 (179-2136) | 695 (332-1992) | 901 (179-2136) | 901 (668-1182) | 900 (179-2136) | 1503 (1254-1752) | 848 (443-1253) | ≤0.05^b^ (0.07^a^) |
| **TCD4- cells** | 594 (142-1975) | 535 (139-1459) | 526 (139-1459) | 460 (252-1011) | 430 (252-1011) | 533 (262-792) | 976 (691-1261) | 586 (543-628) | NS |
| **NK-cells** | 305 (77-1344) | 143 (5.3-1494) | 154 (24-1494) | 71 (5.3-1198) | 239 (72-1198) | 38 (5.3-70) | 332 (157-506) | 321 (43-600) | ≤0.05^a,b,c,e,g^ |
| **B-cells** | 219 (29-710) | 160 (8.6-751) | 125 (86-751) | 177 (8.6-395) | 204 (8.6-312) | 137 (9.6-395) | 466 (279-653) | 176 (124-227) | NS (0.075^a^) |
| **ILC** | 1.2 (0-5.2) | 1.5 (0-3.7) | 1.2 (0-3.5) | 1.5 (0-3.7) | 2.3 (0-3.7) | 1.5 (0-1.7) | 2.1 (1.4-2.8) | 2.2 (0.82-3.5) | NS |
| **ILC2** | 0.48 (0.00-2.9) | 0.43 (0.00-1.8) | 0.33 (0.00-1.8) | 0.43 (0.00-1.8) | 0.43 (0.00-1.8) | 0.43 (0.00-1.1) | 1.3 (0.94-1.7) | 0.82 (0.33-1.3) | NS |
| **ILC3** | 0.77 (0.00-3.1) | 0.86 (0.00-2.3) | 0.89 (0.00-2.1) | 0.76 (0.00-2.3) | 1.9 (0.00-2.3) | 0.74 (0.00-1.1) | 0.80 (0.50-1.1) | 1.3 (0.49-2.2) | NS |
| **Tumor cells** | NA | 673 (17-9521) | 1388 (49-9521) | 598 (83-5466) | 278 (83-733) | 3094 (397-5466) | 81 (17-145) | 240 (36-443) | ≤0.05^e^ |

**Supplementary Table 5 |** Distribution of normal/residual PB leukocyte subsets in T-LGLL and their major TCD4+ and TCD8+ T DP and Tγδ subgroups.

Results expressed as median (range) number of cells/ μl.

^a^ Total T-LGLL *vs.* HD; ^b^ TαβCD4+ LGLL *vs.* HD, ^c^ TαβCD8+ LGLL *vs.* HD, ^d^ TαβCD4+ LGLL *vs.* TαβCD8+ LGLL, ^e^ TαβCD8+ LGLL *STAT3 WT* *vs.* TαβCD8+ LGLL *STAT3* mutated, ^f^ TαβCD8+ LGLL *STAT3 WT* *vs.* HD, ^g^ TαβCD8+ LGLL *STAT3* mutated *vs.* HD.

Abbreviations (alphabetical order): DP, double (CD4+CD8+) positive; HD, healthy donor; ILC, innate lymphoid cell; NA, not applicable; NS, differences not statistically significant; PB, peripheral blood; T-LGLL, T-large granular lymphocytic leukemia; WT, wild type

**Supplementary Table 6 |** Distribution of normal/residual PB Th subsets in total T-PLL and their TCD4+, TCD8+ and TCD4+CD8+ subgroups.

|  | **HD**  **(n=37)** | **Total T-PLL**  **(n=14)** | **T-PLL subtypes** | | | **P-value** |
| --- | --- | --- | --- | --- | --- | --- |
|  |  |  | **TCD4+ PLL (n=8)** | **TCD8+ PLL (n=2)** | **DP T-PLL**  **(n=4)** |  |
| ***Classical TCD4+ cell subsets*** |  |  |  |  |  |  |
| Naive | 316 (37-933) | 0.75 (0-190) | 0 (0-12) | 25 (12-37) | 2.7 (0-190) | ≤0.01^a,b,c^ |
| Th1 | 119 (38-497) | 82 (0-422) | 131 (0-422) | 23 (11-35) | 58 (11-92) | ≤0.05^c^ |
| Th2 | 45 (14-192) | 17 (0-73) | 8.6 (0-73) | 35 (26-45) | 23 (4.7-37) | ≤0.05^a,b,c^ |
| Th17 | 28 (13-126) | 16 (0-2014) | 12 (0-71) | 1016 (19-2014) | 14 (4.2-28) | ≤0.05^a,b,c^ |
| Th1/17 | 59 (21-241) | 24 (0-69) | 21 (0-69) | 38 (29-46) | 17 (1.5-33) | ≤0.05^a,b,c^ |
| Th22 | 11 (1.4-43) | 3.1 (0-42) | 5.9 (0-42) | 0.65 (0-1.3) | 4.3 (1.4-9.2) | ≤0.05^a,c^ |
| ***Non-classical TCD4+ cell subsets*** |  |  |  |  |  |  |
| CD183^+^CD194^+^CD196^+^CCR10^+^ | 6.3 (0.52-23) | 0.95 (0-20) | 3.6 (0-20) | 0 (0-0) | 1.1 (0-4.5) | ≤0.05^a,c^ |
| CD183^+^CD194^+^CD196^+^CCR10^-^ | 30 (1.2-124) | 17 (0-145) | 11 (0-145) | 28 (23-33) | 17 (5.2-38) | ≤0.05^a^ (0.06^b^) |
| CD183^+^CD194^+^CD196^-^CCR10^+^ | 5.5 (0.55-14) | 2.1 (0-29) | 2.6 (0-29) | 0 (0-0) | 2.4 (0-6.2) | ≤0.05^a^ (0.09^c^) |
| CD183^+^CD194^+^CD196^-^CCR10^-^ | 43 (6.2-122) | 17 (0-150) | 6.7 (0-150) | 36 (12-59) | 28 (7.2-47) | ≤0.05^a,b^ |
| CD183^+^CD194^-^CD196^+^CCR10^+^ | 0.40 (0-3.7) | 0 (0-11) | 0 (0-11) | 0 (0-0) | 0 (0-0) | ≤0.05^a,c^ |
| CD183^+^CD194^-^CD196^-^CCR10^+^ | 0.50 (0-2.9) | 0 (0-15) | 0.70 (0-15) | 0 (0-0) | 0 (0-0) | ≤0.05^a,c^ |
| CD183^-^CD194^-^CD196^+^CCR10^-^ | 7.8 (0-44) | 3.2 (0-497) | 0 (0-8.2) | 249 (1.9-497) | 5.4 (0-8.7) | ≤0.05^a,b^ |
| CD183^-^CD194^+^CD196^-^CCR10^+^ | 4.7 (0.39-56) | 0.44 (0-27) | 0 (0-27) | 0 (0-0) | 1.7 (0.87-5.7) | ≤0.05^a,b^ (0.07^c^) |
| CD183^-^CD194^-^CD196^-^CCR10^-^ | 3.1 (0-18) | 0 (0-32) | 0 (0-32) | 1.4 (0-2.8) | 0.80 (0-18) | ≤0.05^a,b^ |

Results expressed as median (range) number of cells/ μl.

^a^ Total T-PLL *vs.* HD; ^b^ TCD4+ PLL *vs.* HD, ^c^ DP T-PLL *vs.* HD, ^d^ DP T-PLL *vs.* TCD4+ PLL.

Abbreviations (alphabetical order): DP, double (CD4+CD8+) positive; HD, healthy donor; NS, differences not statistically significant; PB, peripheral blood; T-PLL, T-prolymphocytic leukemia.

**Supplementary Table 7 |** Distribution of normal/residual PB TFH and Treg cell subsets in T-PLL and their TCD4+, TCD8+ and TCD4+CD8+ subgroups.

|  | **HD**  **(n=37)** | **Total T-PLL**  **(n=14)** | **T-PLL subtypes** | | | **P-value** |
| --- | --- | --- | --- | --- | --- | --- |
|  |  |  | **CD4+ T-PLL (n=8)** | **CD8+ T-PLL (n=2)** | **DP T-PLL**  **(n=4)** |  |
| **Follicular Helper T (TFH) cells** | 129 (34-277) | 71 (0-280) | 60 (0-280) | 97 (92-102) | 77 (13-140) | ≤0.01^a,b^ |
| Treg-like | 14 (0-52) | 6.6 (0-81) | 2.0 (0-81) | 24 (11-37) | 7.6 (4.5-23) | ≤0.05^a,b^ |
| Naive-like | 10 (0-25) | 0 (0-14) | 0 (0-6.9) | 1.9 (0-3.7) | 0.90 (0-14) | ≤0.05^a,b,c^ |
| Th1-like | 21 (0-48) | 11 (0-29) | 11 (0-29) | 8.4 (5.6-11) | 14 (0.65-19) | ≤0.05^a,b^ (0.08^c^) |
| Th2-like | 9.5 (0-19) | 3.1 (0-36) | 1.9 (0-36) | 4.7 (0-9.3) | 8.4 (0.58-22) | ≤0.05^a,b^ |
| Th17-like | 23 (0-55) | 10 (0-36) | 6.2 (0-36) | 17 (16-19) | 11 (1.8-22) | ≤0.05^a,b,c^ |
| Th1/17-like | 6.6 (0-15) | 2.2 (0-11) | 2.1 (0-11) | 0.95 (0-1.9) | 2.7 (0-3.2) | ≤0.05^a,b,c^ |
| CD183^+^CD194^+^CD196^+^CCR10^-^-like | 11 (0-28) | 4.3 (0-23) | 5.2 (0-23) | 2.4 (0-4.8) | 3.6 (1.3-8.1) | ≤0.05^a,c^ |
| CD183^+^CD194^+^CD196^-^CCR10^-^-like | 15 (0-57) | 7.7 (0-44) | 7.2 (0-44) | 14 (6.4-22) | 11 (3.5-25) | NS |
| CD183^-^CD194^-^CD196^+^CCR10^-^-like | 11 (0-34) | 3.4 (0-14) | 2.6 (0-14) | 8.1 (3.0-13) | 3.6 (0.31-9.9) | ≤0.05^a,b,c^ |
| CD183^-^CD194^-^CD196^-^CCR10^-^-like | 3.8 (0-23) | 1.9 (0-11) | 1.4 (0-10.0) | 1.4 (0-2.7) | 5.2 (0.72-11) | NS (0.07^b^) |
| **Regulatory T (Treg) cells** | 49 (20-156) | 34 (0-256) | 24 (0-256) | 73 (61-85) | 41 (10-84) | NS (0.08^b^) |
| Naive-like | 5.9 (0.71-31) | 1.7 (0-13) | 0.80 (0-8.4) | 6.6 (0-13) | 2.4 (0-5.6) | ≤0.01^a,b^ (0.06^c^) |
| Th1-like | 3.1 (0.91-14) | 2.2 (0-8.9) | 0.70 (0-7.9) | 2.2 (0-4.4) | 3.0 (0-8.9) | NS |
| Th2-like | 4.3 (1.5-20) | 1.9 (0-12) | 0.85 (0-12) | 4.1 (0-8.1) | 3.3 (0.72-12) | ≤0.05^a,b^ |
| Th17-like | 8.6 (3.5-30) | 4.4 (0-16) | 4.0 (0-16) | 4.4 (0-8.8) | 7.2 (1.1-12) | ≤0.05^a,b^ |
| Th22-like | 5.4 (0.77-19) | 3.5 (0-18) | 3.1 (0-18) | 1.9 (0-3.7) | 5.7 (1.7-7.4) | NS |
| CD183^+^CD194^+^CD196^+^CCR10^+^-like | 3.2 (1.1-15) | 1.4 (0-13) | 1.6 (0-13) | 0 (0-0) | 2.7 (1.0-4.6) | ≤0.05^a,b^ |
| CD183^+^CD194^+^CD196^+^CCR10^-^-like | 7.4 (3.2-37) | 3.3 (0-103) | 3.2 (0-103) | 18 (7.5-28) | 5.4 (1.3-11) | ≤0.05^b^ (0.07^a^) |
| CD183^+^CD194^+^CD196^-^CCR10^+^-like | 1.0 (0-7.1) | 0.67 (0-10) | 1.1 (0-10) | 0 (0-0) | 1.8 (0.43-7.1) | NS |
| CD183^+^CD194^+^CD196^-^CCR10^-^-like | 3.8 (1.00-19) | 4.7 (0-86) | 2.8 (0-86) | 11 (11-11) | 4.7 (1.3-15) | NS |
| CD183^-^CD194^+^CD196^-^CCR10^+^-like | 1.00 (0-6.5) | 0.30 (0-9.6) | 0 (0-9.6) | 0.60 (0-1.2) | 1.1 (0.60-5.7) | ≤0.05^b^ (0.06^a^) |

Results expressed as median (range) number of cells/ μl. ^a^ Total T-PLL *vs.* HD; ^b^ TCD4+ PLL *vs.* HD, ^c^ DP T-PLL *vs.* HD, ^d^ DP T-PLL *vs.* TCD4+ PLL.

Abbreviations (alphabetical order): DP, double (CD4+CD8+) positive; HD, healthy donor; NS, differences not statistically significant; PB, peripheral blood; T-PLL, T-prolymphocytic leukemia.

**Supplementary Table 8 |** Distribution of normal/residual PB Th subset cells in SS/MF patients.

|  | **HD**  **(n=21)** | **SS/MF**  **(n=7)** | **P-value** |
| --- | --- | --- | --- |
|  |  |  |  |
| ***Classical TCD4+ cell subsets*** |  |  |  |
| Naive | 302 (84-1432) | 121 (10-222) | ≤0.01 |
| Th1 | 119 (39-704) | 44 (26-493) | NS |
| Th2 | 44 (13-92) | 15 (2.5-66) | ≤0.01 |
| Th17 | 37 (5.7-87) | 33 (5.0-80) | NS |
| Th1/17 | 38 (4.3-151) | 15 (2.9-144) | NS |
| Th22 | 9.0 (2.7-34) | 19 (0.75-52) | NS |
| ***Non-classical TCD4+ cell subsets*** |  |  |  |
| CD183^+^CD194^+^CD196^+^CCR10^+^ | 7.3 (0-40) | 6.7 (0.75-88) | NS |
| CD183^+^CD194^+^CD196^+^CCR10^-^ | 39 (5.6-123) | 27 (4.9-55) | NS (0.095) |
| CD183^+^CD194^+^CD196^-^CCR10^+^ | 6.4 (0.42-22) | 6.7 (0-27) | NS |
| CD183^+^CD194^+^CD196^-^CCR10^-^ | 47 (4.8-115) | 16 (5.4-86) | ≤0.05 |
| CD183^+^CD194^-^CD196^+^CCR10^+^ | 0.50 (0-1.9) | 0.26 (0-1.3) | NS |
| CD183^+^CD194^-^CD196^-^CCR10^+^ | 0.30 (0-1.7) | 0 (0-2.5) | NS |
| CD183^-^CD194^-^CD196^+^CCR10^-^ | 7.1 (0-15) | 0.78 (0-9.6) | NS |
| CD183^-^CD194^+^CD196^-^CCR10^+^ | 6.1 (0.87-103) | 4.0 (0.53-57) | NS |
| CD183^-^CD194^-^CD196^-^CCR10^-^ | 2.0 (0-10) | 0 (0-5.9) | NS |

Results expressed as median (range) number of cells/ μl.

Abbreviations (alphabetical order): HD, healthy donor; MF, mycosis fungoides; NS, differences not statistically significant; SS, Sezary syndrome; PB, peripheral blood.

**Supplementary Table 9 |** Distribution of normal/residual PB TFH and Treg cell subsets in SS/MF patients.

|  | **HD**  **(n=21)** | **SS/MF**  **(n=7)** | **P-value** |
| --- | --- | --- | --- |
|  |  |  |  |
| **Follicular Helper T (TFH) cells** | 111 (34-241) | 53 (11-206) | ≤0.05 |
| Treg-like | 13 (3.2-56) | 11 (2.2-31) | NS |
| Naive-like | 9.8 (2.2-32) | 3.6 (0.89-19) | NS |
| Th1-like | 14 (2.5-39) | 4.2 (1.3-27) | NS (0.06) |
| Th2-like | 11 (4.9-27) | 3.9 (0.26-14) | ≤0.05 |
| Th17-like | 23 (0.64-49) | 14 (2.0-43) | NS |
| Th1/17-like | 3.7 (0-18) | 2.2 (0.41-12) | NS |
| CD183^+^CD194^+^CD196^+^CCR10^-^-like | 7.8 (0-45) | 3.8 (0.77-13) | ≤0.05 |
| CD183^+^CD194^+^CD196^-^CCR10^-^-like | 15 (5.3-38) | 3.8 (0.85-20) | ≤0.01 |
| CD183^-^CD194^-^CD196^+^CCR10^-^-like | 6.6 (0-26) | 2.7 (1.2-23) | NS |
| CD183^-^CD194^-^CD196^-^CCR10^-^-like | 1.1 (0-15) | 2.8 (0.37-25) | ≤0.05 |
| **Regulatory T (Treg) cells** | 53 (20-142) | 27 (14-184) | NS |
| Naive-like | 6.2 (1.3-33) | 4.1 (0-14) | NS |
| Th1-like | 2.3 (0.40-16) | 1.2 (0-8.5) | NS |
| Th2-like | 4.6 (1.6-9.6) | 1.3 (0.84-14) | NS (0.08) |
| Th17-like | 8.8 (0-20) | 6.8 (1.4-11) | NS |
| Th22-like | 5.6 (0-11) | 2.6 (0.81-33) | NS |
| CD183^+^CD194^+^CD196^+^CCR10^+^-like | 3.7 (0-9.4) | 2.2 (0.59-97) | NS |
| CD183^+^CD194^+^CD196^+^CCR10^-^-like | 5.9 (0-25) | 3.3 (0.83-17) | NS |
| CD183^+^CD194^+^CD196^-^CCR10^+^-like | 1.1 (0-8.8) | 1.3 (0-9.9) | NS |
| CD183^+^CD194^+^CD196^-^CCR10^-^-like | 4.4 (1.7-26) | 2.0 (0.90-9.9) | ≤0.05 |
| CD183^-^CD194^+^CD196^-^CCR10^+^-like | 0.80 (0-3.4) | 1.4 (0.44-6.3) | NS |

Results expressed as median (range) number of cells/ μl.

Abbreviations (alphabetical order): HD, healthy donor; MF, mycosis fungoides; NS, differences not statistically significant; SS, Sezary syndrome; PB, peripheral blood.

**Supplementary Table 10 |** Distribution of normal/residual PB Th cell subsets in T-LGLL and their major TCD4+ and TCD8+ T DP and Tγδ subgroups.

|  | **HD**  **(n=72)** | **Total**  **T-LGLL**  **(n=26)** | **T-LGLL subtypes** | | | | | | **P-value** |
| --- | --- | --- | --- | --- | --- | --- | --- | --- | --- |
|  |  |  | **TαβCD4+ (n=12)** | **TαβCD8+ (n=10)** | **TαβCD8+ *STAT3* WT**  **(n=5)** | **TαβCD8+ *STAT3* mutated**  **(n=5)** | **TαβDP**  **(n=2)** | **Tγδ**  **(n=2)** |  |
| ***Classical TCD4+ cell subsets*** |  |  |  |  |  |  |  |  |  |
| Naive | 343 (25-1017) | 231 (1.1-874) | 192 (20-443) | 234 (1.1-874) | 241 (86-368) | 220 (1.1-874) | 516 (338-694) | 248 (149-346) | ≤0.05^a,b^ |
| Th1 | 125 (27-704) | 113 (3.1-418) | 95 (20-197) | 152 (3.1-418) | 208 (30-418) | 142 (3.1-195) | 148 (134-161) | 96 (57-136) | NS (0.06^b^) |
| Th2 | 52 (13-210) | 36 (8.8-418) | 26 (11-114) | 46 (8.8-418) | 41 (22-418) | 51 (8.8-61) | 75 (49-101) | 44 (28-59) | ≤0.05^a,b^ |
| Th17 | 44 (13-152) | 38 (9.1-345) | 25 (12-94) | 42 (9.1-345) | 70 (22-345) | 38 (9.1-43) | 49 (46-52) | 26 (16-36) | ≤0.05^l^ (0.06^b^) |
| Th1/17 | 74 (5.2-394) | 76 (1.2-491) | 67 (13-130) | 89 (1.2-491) | 103 (35-491) | 76 (1.2-141) | 121 (113-128) | 99 (31-167) | NS |
| Th22 | 12 (2.6-85) | 12 (0-173) | 9.7 (0-47) | 13 (0.74-173) | 19 (4.1-173) | 10 (0.74-25) | 20 (17-24) | 8.3 (4.4-12) | NS |
| ***Non-classical TCD4+ cell subsets*** |  |  |  |  |  |  |  |  |  |
| CD183^+^CD194^+^CD196^+^CCR10^+^ | 6.4 (0.30-36) | 12 (0.86-164) | 9.8 (0.86-32) | 11 (1.4-164) | 13 (6.1-164) | 6.9 (1.4-27) | 22 (18-26) | 12 (1.8-22) | ≤0.05^a^ |
| CD183^+^CD194^+^CD196^+^CCR10^-^ | 39 (8.0-200) | 52 (14-485) | 44 (17-184) | 54 (14-485) | 57 (14-485) | 40 (19-134) | 97 (95-99) | 43 (15-71) | NS |
| CD183^+^CD194^+^CD196^-^CCR10^+^ | 5.4 (0.12-20) | 7.2 (1.4-84) | 6.3 (1.6-56) | 6.8 (1.4-84) | 6.8 (2.0-84) | 4.1 (1.4-20) | 18 (16-20) | 9.0 (3.5-14) | NS |
| CD183^+^CD194^+^CD196^-^CCR10^-^ | 51 (6.2-251) | 58 (18-496) | 55 (22-212) | 51 (18-496) | 42 (18-496) | 60 (21-111) | 88 (84-92) | 60 (35-84) | NS |
| CD183^+^CD194^-^CD196^+^CCR10^+^ | 0.30 (0-6.3) | 0.02 (0-73) | 0.02 (0-7.0) | 0.45 (0-73) | 2.2 (0-73) | 0 (0-1.3) | 1.4 (0-2.7) | 0.55 (0-1.1) | NS |
| CD183^+^CD194^-^CD196^-^CCR10^+^ | 0.51 (0-3.9) | 0.24 (0-76) | 0.90 (0-6.3) | 0 (0-76) | 0.48 (0-76) | 0 (0-2.2) | 2.6 (0-5.1) | 0.47 (0-0.94) | NS |
| CD183^-^CD194^-^CD196^+^CCR10^-^ | 8.3 (0-44) | 2.1 (0-31) | 2.4 (1.2-9.2) | 3.6 (0-31) | 8.1 (1.6-31) | 0 (0-7.9) | 0 (0-0) | 1.2 (0.78-1.6) | ≤0.05^a,b,e^(0.08^c^) |
| CD183^-^CD194^+^CD196^-^CCR10^+^ | 6.0 (0.87-34) | 8.3 (0.57-107) | 6.1 (0.57-45) | 9.4 (1.8-107) | 12 (4.9-107) | 8.3 (1.8-10) | 9.1 (8.2-9.9) | 5.8 (5.2-6.4) | NS |
| CD183^-^CD194^-^CD196^-^CCR10^-^ | 3.1 (0-15) | 1.5 (0-25) | 2.6 (0.46-10.0) | 1.9 (0-25) | 5.7 (0-25) | 1.4 (0-3.0) | 0 (0-0) | 1.4 (0.68-2.1) | NS |

Results expressed as median (range) number of cells/ μl.

^a^ Total T-LGLL *vs.* HD; ^b^ TαβCD4+ LGLL *vs.* HD, ^c^ TαβCD8+ LGLL *vs.* HD, ^d^ TαβCD4+ LGLL *vs.* TαβCD8+ LGLL, ^e^ TαβCD8+ LGLL *STAT3 WT* *vs.* TαβCD8+ LGLL *STAT3* mutated, ^f^ TαβCD8+ LGLL *STAT3 WT* *vs.* HD, ^g^ TαβCD8+ LGLL *STAT3* mutated *vs.* HD.

Abbreviations (alphabetical order): DP, double (CD4+CD8+) positive; HD, healthy donor; NS, differences not statistically significant; PB, peripheral blood; Th, T helper cells; T-LGLL, T-large granular lymphocytic leukemia; WT, wild type.

**Supplementary Table 11 |** Distribution of normal/residual PB TFH and Treg subsets in T-LGLL and major subtypes.

|  | **HD**  **(n=72)** | **Total T-LGLL**  **(n=26)** | **T-LGLL subtypes** | | | | | | **P-value** |  |
| --- | --- | --- | --- | --- | --- | --- | --- | --- | --- | --- |
|  |  |  | **TαβCD4+ (n=12)** | **TαβCD8+ (n=10)** | **TαβCD8+ *STAT3* WT**  **(n=5)** | **TαβCD8+ *STAT3* mutated**  **(n=5)** | **TαβDP**  **(n=2)** | **Tγδ**  **(n=2)** |  |  |
| **Follicular Helper T (TFH) cells** | | 124 (22-483) | 140 (43-519) | 108 (43-519) | 140 (47-465) | 147 (47-176) | 132 (49-465) | 271 (196-345) | 157 (65-249) | NS |
| Treg-like | | 13 (0-42) | 17 (2.8-312) | 14 (3.7-67) | 16 (2.8-312) | 17 (2.8-312) | 13 (7.9-63) | 52 (32-72) | 18 (7.2-28) | NS (0.06^a^) |
| Naive-like | | 8.8 (0-54) | 2.9 (0-112) | 2.6 (0-20) | 2.9 (0-112) | 4.0 (0.99-112) | 1.3 (0-37) | 11 (4.1-18) | 4.9 (0.47-9.4) | ≤0.05^a,b,g^ |
| Th1-like | | 17 (0-88) | 13 (0.58-224) | 11 (3.1-41) | 13 (0.58-224) | 13 (12-224) | 15 (0.58-50) | 27 (19-35) | 24 (11-36) | NS |
| Th2-like | | 9.7 (0-52) | 11 (0.81-143) | 9.8 (2.5-105) | 11 (0.81-143) | 10 (3.1-143) | 11 (0.81-49) | 23 (12-34) | 14 (8.6-19) | NS |
| Th17-like | | 27 (0-84) | 28 (4.0-321) | 18 (7.1-120) | 32 (4.0-321) | 44 (4.0-321) | 27 (4.4-57) | 48 (43-53) | 35 (9.1-60) | NS |
| Th1/17-like | | 6.3 (0-74) | 5.8 (0-84) | 3.5 (0.90-13) | 6.9 (0-84) | 7.6 (2.6-84) | 6.2 (0-34) | 8.1 (7.2-8.9) | 7.6 (4.1-11) | NS (0.07^b^) |
| CD183^+^CD194^+^CD196^+^CCR10^-^-like | | 9.2 (0-44) | 14 (0-177) | 12 (2.9-40) | 14 (0-177) | 16 (2.0-177) | 13 (0-67) | 31 (26-36) | 18 (3.5-32) | NS |
| CD183^+^CD194^+^CD196^-^CCR10^-^-like | | 12 (0-57) | 20 (1.5-291) | 16 (4.6-100) | 22 (1.5-291) | 17 (1.5-291) | 28 (13-79) | 49 (31-67) | 25 (11-40) | ≤0.05^a,,c,g^ |
| CD183^-^CD194^-^CD196^+^CCR10^-^-like | | 11 (0-61) | 6.2 (0-46) | 4.0 (0-21) | 8.4 (0-46) | 6.1 (4.6-46) | 11 (0-13) | 11 (9.9-11) | 6.2 (3.2-9.2) | ≤0.01^a,b^ |
| CD183^-^CD194^-^CD196^-^CCR10^-^-like | | 2.2 (0-19) | 6.4 (0.60-45) | 5.5 (1.6-23) | 5.9 (0.60-45) | 3.8 (2.3-45) | 11 (0.60-17) | 12 (12-12) | 4.8 (3.0-6.5) | ≤0.05^a,b,c^ |
| **Regulatory T (Treg) cells** | | 63 (21-178) | 44 (8.3-408) | 37 (20-110) | 45 (8.3-408) | 45 (32-408) | 42 (8.3-119) | 67 (65-68) | 37 (31-44) | ≤0.05^a,b^ |
| Naive-like | | 9.1 (1.1-72) | 3.6 (0-75) | 3.5 (0-21) | 5.2 (0-75) | 9.4 (1.5-75) | 3.0 (0-8.5) | 11 (8.1-13) | 4.6 (3.0-6.2) | ≤0.05^a,b,g^(0.09^e^) |
| Th1-like | | 3.4 (0.17-14) | 2.8 (0-17) | 2.8 (0-8.4) | 2.9 (0.53-17) | 1.6 (1.3-17) | 4.0 (0.53-7.7) | 3.0 (2.7-3.3) | 2.4 (1.5-3.3) | ≤0.05^e^ (0.09^a,b^) |
| Th2-like | | 4.3 (0.62-22) | 3.9 (1.3-52) | 4.5 (1.3-19) | 3.4 (2.0-52) | 3.0 (2.1-52) | 3.7 (2.0-10) | 3.5 (2.8-4.1) | 3.8 (2.2-5.3) | NS |
| Th17-like | | 11 (3.5-53) | 8.6 (1.4-49) | 6.9 (2.4-24) | 8.9 (1.4-49) | 7.8 (6.1-49) | 9.9 (1.4-24) | 12 (11-14) | 6.3 (3.6-8.9) | ≤0.05^a,b,e^ |
| Th22-like | | 6.5 (1.5-24) | 5.6 (0-38) | 5.6 (0-14) | 5.9 (0-38) | 7.6 (2.9-38) | 5.4 (0-9.8) | 6.1 (3.8-8.3) | 2.9 (2.3-3.4) | NS |
| CD183^+^CD194^+^CD196^+^CCR10^+^-like | | 3.2 (0.12-17) | 2.7 (0-16) | 2.3 (0-6.7) | 3.7 (0-16) | 3.8 (2.6-16) | 1.4 (0-13) | 5.2 (4.4-5.9) | 1.6 (1.5-1.7) | NS (0.08^a^) |
| CD183^+^CD194^+^CD196^+^CCR10^-^-like | | 10.0 (2.2-36) | 7.4 (0-26) | 6.3 (1.7-19) | 6.7 (0-26) | 6.2 (0-19) | 8.0 (1.4-26) | 12 (11-14) | 7.3 (3.6-11) | ≤0.05^b^ (0.06^a^) |
| CD183^+^CD194^+^CD196^-^CCR10^+^-like | | 1.1 (0-7.3) | 1.4 (0-12) | 1.2 (0-4.8) | 1.4 (0-12) | 1.4 (1.2-12) | 0 (0-3.9) | 2.6 (2.4-2.8) | 1.1 (0.74-1.4) | NS |
| CD183^+^CD194^+^CD196^-^CCR10^-^-like | | 4.3 (0.80-23) | 4.7 (1.3-62) | 3.4 (1.4-11) | 4.6 (1.3-62) | 2.7 (2.4-62) | 4.8 (1.3-14) | 8.3 (8.2-8.3) | 4.9 (4.5-5.2) | NS |
| CD183^-^CD194^+^CD196^-^CCR10^+^-like | | 1.2 (0-13) | 1.3 (0-11) | 1.3 (0.42-9.5) | 1.2 (0-11) | 1.4 (0.95-11) | 0.94 (0-3.9) | 2.4 (2.1-2.7) | 2.1 (0.56-3.7) | NS |

Results expressed as median (minimum – maximum value) of cells/ μl.

^a^ Total T-LGLL *vs.* HD; ^b^ TαβCD4+ LGLL *vs.* HD, ^c^ TαβCD8+ LGLL *vs.* HD, ^d^ TαβCD4+ LGLL *vs.* TαβCD8+ LGLL, ^e^ TαβCD8+ LGLL *STAT3 WT* *vs.* TαβCD8+ LGLL *STAT3* mutated, ^f^ TαβCD8+ LGLL *STAT3 WT* *vs.* HD, ^g^ TαβCD8+ LGLL *STAT3* mutated *vs.* HD.

Abbreviations (alphabetical order): DP, double positive (CD4+CD8+); HD, healthy donor; NS, differences not statistically significant; PB, peripheral blood; TFH, T follicular helper cells; T-LGLL, T-large granular lymphocytic leukemia; Treg, regulatory T cells; WT, wild type.

**SUPPLEMENTARY FIGURES**

**Supplementary Figure 1**

**Monocytes**

**Total ILC**

**Normalized cell counts (percentile)**

**ILC2**

**ILC3**

**NK-cells**

**Normalized cell counts (percentile)**

**Normalized cell counts (percentile)**

**Normalized cell counts (percentile)**

**Distribution of major normal leukocyte populations in blood of T-PLL patients grouped according to the progression status of the disease.** Box plots show the distribution of different WBC populations in blood expressed in terms of cells/µL for T-PLL cases, grouped depending on the occurrence or not of disease progression normalized by the corresponding age-matched HD. Data is shown only for those major cell populations for which statistically significant differences were observed between the groups. In all graphs, dots correspond to individual samples, while notched boxes represent the 25^th^ and 75^th^ values; the lines inside the box correspond to median values (50^th^ percentile=0) and whiskers represent minimum and maximum values. Gray dots represent HD subjects and dark blue dots denote T-PLL cases. *p-value ≤0.05, **p-value ≤0.01 and ***p-value ≤0.001 *vs*. HD. P-values resulting from comparisons between patients without and with progression are depicted as ^#^p-value ≤0.05; trend between T-PLL groups without and with progression is shown in brackets as p-value = 0.06. The gray shading corresponds to the 5^th^-95^th^ percentiles (values -45 and 45, respectively).

Abbreviations (alphabetical order): HD, healthy donor; ILC, innate lymphoid cell; NK, natural-killer; T-PLL, T-prolymphocytic leukemia; WBC: white blood cells.

**Supplementary Figure 2**

**TCD4+ cells**

**T-cells**

**NK-cells**

**Dendritic cells**

**Normal WBC**

**Normalized cell counts (percentile)**

**Normalized cell counts (percentile)**

**Normalized cell counts (percentile)**

**Normalized cell counts (percentile)**

**Normalized cell counts (percentile)**

**Total ILC**

**Total ILC**

**ILC2**

**ILC3**

**ILC3**

**ILC2**

**Normalized cell counts (percentile)**

**Normalized cell counts (percentile)**

**Normalized cell counts (percentile)**

**Normalized cell counts (percentile)**

**Distribution of major normal leukocyte populations in blood of T-PLL patients grouped according to the maturational-related and functional-associated stage of their tumor cells.** Box plots show the distribution of normalized counts of different WBC populations in blood (percentile values relative to age-matched HD data reference ranges) for T-PLL cases grouped according to the tumor cell maturation-associated (early maturation stage -naive-naive/CM- *vs*. memory stage -CM-TM-) and Th-related (one single *vs*. ≥2) profiles, compared to HD. Data is shown only for those major cell populations for which statistically significant differences were observed among the groups. In all graphs, dots correspond to individual samples, while notched boxes represent the 25^th^ and 75^th^ percentile values; the lines inside the box correspond to median values (50^th^ percentile=0) and whiskers represent minimum and maximum values. Gray dots represent HD subjects and dark blue dots denote T-PLL cases. *p-value ≤0.05, **p-value ≤0.01 and ***p-value ≤0.001 *vs*. HD. P-values resulting from comparisons between maturation-related or Th-associated phenotypes are depicted as ^#^p-value ≤0.05. Trends *vs*. HD or between the T-PLL groups of patients are depicted with p-values <0.1. The gray shading corresponds to the 5^th^-95^th^ percentiles (values -45 and 45, respectively).

Abbreviations (alphabetical order): CM, central memory; HD, healthy donor; N, naive; NK, natural-killer; ns, differences not statistically significant; TM, transitional memory; T-PLL, T-prolymphocytic leukemia; WBC: white blood cells.

**Supplementary Figure 3**

**T-cells**

**Lymphocytes**

**Dendritic cells**

**Neutrophils**

**Normalized cell counts (percentile)**

**Normalized cell counts (percentile)**

**Normalized cell counts (percentile)**

**Normalized cell counts (percentile)**

SS/MF

SS/MF

SS/MF

SS/MF

**Distribution of major/normal leukocyte populations in blood of patients with SS/MF grouped according to the functional-associated stage of their tumor cells.** Box plots show the distribution of normalized counts of the different populations of WBC (percentile values relative to age-matched HD data reference ranges) for SS/MF cases, grouped according to their Th-related (Th2 *vs*. Th17) tumor cell profile, compared to HD. Data is shown only for those major cell populations for which statistically significant differences were observed among the groups. In all graphs, dots correspond to individual samples, while notched boxes represent the 25^th^ and 75^th^ percentile values; the lines inside the box correspond to median values (50^th^ percentile=0) and whiskers represent minimum and maximum values. Gray dots depict HD subjects and pink and black dots SS and MF patients, respectively. *p-value ≤0.05 and **p-value ≤0.01 *vs*. HD. P-values resulting from comparisons between groups of cases with different Th-associated phenotypes are depicted as ^#^p-value ≤0.05. Trends *vs*. HD are depicted with p-values <0.1. The gray shading corresponds to the 5^th^-95^th^ percentiles (values -45 and 45, respectively).

Abbreviations (alphabetical order): HD, healthy donor; MF, Mycosis fungoides; ns, differences not statistically significant; SS, Sézary syndrome; Th, T helper.

**Supplementary Figure 4**

**Normal WBC**

**B-cells**

**NK-cells**

**Neutrophils**

**Basophils**

**Normalized cell counts (percentile)**

**Normalized cell counts (percentile)**

**Normalized cell counts (percentile)**

**Normalized cell counts (percentile)**

**Normalized cell counts (percentile)**

**Normalized cell counts (percentile)**

**Normalized cell counts (percentile)**

**Distribution of major normal leukocyte populations in blood of patients with TαβCD8+** **LGLL grouped according to the functional-associated phenotypic profile of their clonal T-cells.** Box plots show the distribution of normalized counts of the different populations of WBC (percentile values relative to age-matched HD data reference ranges) for T-LGLL cases grouped according to their Th-related (Th1 *vs*. CR-) profile in TαβCD8+ clonal T-cell, compared to HD. Data is shown only for those major cell populations for which statistically significant differences were observed. In all graphs, dots correspond to individual samples, while notched boxes represent the 25^th^ and 75^th^ percentile values; the lines inside the box correspond to median values (50^th^ percentile=0) and whiskers represent minimum and maximum values. Gray dots represent HD subjects and orange dots correspond to TαβCD8+ LGLL cases. *p-value ≤0.05 and ***p-value ≤0.001 *vs*. HD. P-values resulting from comparisons between groups of cases with different Th-associated phenotypes are depicted as ^#^p-value ≤0.05. Trend for comparations between the groups is depicted as p-value = 0.07. The gray shading corresponds to the 5^th^-95^th^ percentiles (values -45 and 45, respectively).

Abbreviations (alphabetical order): CR-, chemokine receptor negative; HD, healthy donor; NK, natural-killer; ns, differences not statistically significant; Th, T helper; T-LGLL, T-large granular lymphocytic leukemia; WBC, white blood cells.

**Supplementary Figure 5**

**T-PLL**

**TFH**

**Th17**

**Th22**

**Normalized Th22 cell counts (percentile)**

**Normalized TFH cell counts (percentile)**

**Normalized TFH cell counts (percentile)**

**Normalized Th22 cell counts (percentile)**

**Normalized Th17 cell counts (percentile)**

**Normalized Th17 cell counts (percentile)**

**T-LGLL**

**SS/MF**

**Treg**

**TFH**

**Normalized Treg cell counts (percentile)**

**Normalized TFH cell counts (percentile)**

SS/MF

**Distribution of major normal TCD4+ cell subsets in blood of T-CLPD patients grouped according to the maturational-related and functional-associated stages of tumor T-cells.** Box plots show the distribution of normalized counts of the different populations of WBC (percentile values relative to age-matched HD data reference ranges) for T-PLL, SS/MF and TαβCD8+ LGLL cases, grouped according to their tumor maturation-associated (early maturation stage -naive-naive/CM- *vs*. memory stage -CM-TM-) profile in T-PLL or Th-related profile in T-PLL, SS/MF and TαβCD8+ LGLL (one *vs*. ≥2, Th2 *vs*. Th17 and Th1 *vs*. CR-, respectively), compared to HD. Data is shown only for those major cell populations for which statistically significant differences were observed. In all graphs, dots correspond to individual samples, while notched boxes represent the 25^th^ and 75^th^ percentile values; the lines inside the box correspond to median values (50^th^ percentile=0) and whiskers represent minimum and maximum values. Gray dots represent HD subjects, dark blue dots denote T-PLL cases, pink and black dots represent SS and MF cases, respectively, and orange dots correspond to the TαβCD8+ LGLL cases. *p-value ≤0.05, **p-value ≤0.01 and ***p-value ≤0.001 *vs*. HD. Trends *vs*. HD are depicted as p-values <0.1. The gray shading corresponds to the 5^th^-95^th^ percentiles (values -45 and 45, respectively).

Abbreviations (alphabetical order): CM, central memory; CR-, chemokine receptor negative; HD, healthy donors; MF, Mycosis fungoides; N, naive; ns, differences not statistically significant; SS, Sézary syndrome; Th, T helper; TM, transitional memory; T-LGLL, T-large granular lymphocytic leukemia; T-PLL, T-prolymphocytic leukemia.

**Supplementary Figure 6**

**Normal residual Th2 cells**

**Normal residual Th1 cells**

**Normalized Th1 cell counts (percentile)**

**Normalized Th2 cell counts (percentile)**

T-cell maturation stage

T-cell maturation stage

T-cell maturation stage

**Normal residual Th1/17 cells**

**Normal residual Th17 cells**

**Normalized Th17 cell counts (percentile)**

**Normalized Th1/17 cell counts (percentile)**

T-cell maturation stage

**Normal residual Th22 cells**

**Normalized Th22 cell counts (percentile)**

T-cell maturation stage

**Distribution per maturation stage of the different Th subsets of TCD4+ cells in blood of T-PLL, SS/MF and both TCD4+ and TCD8+ T-LGLL patients compared to HD**. Box plots show the distribution of normalized counts of the different TCD4+ Th cell populations (percentile values relative to age-matched HD data reference ranges) in blood of HD and T-CLPD patients. In all graphs, dots correspond to individual samples, while notched boxes represent the 25^th^ and 75^th^ percentile values; the lines inside the box correspond to median values (50^th^ percentile=0) and whiskers represent minimum and maximum values. Gray dots represent HD subjects, dark blue dots denote T-PLL cases, pink and black dots represent SS and MF cases, respectively, dark green dots denote total T-LGLL cases, while light green, and orange dots correspond to the TCD4+ and TCD8+ LGLL subtypes, respectively. *p-value ≤0.05, **p-value ≤0.01 and ***p-value ≤0.001 vs. HD. Trends versus HD are depicted as p-values ≤0.1. The gray shading corresponds to the 5^th^-95^th^ percentiles (values -45 and 45, respectively).

Abbreviations (alphabetical order): CM, central memory; HD, healthy donors; EM, effector memory; MF, Mycosis fungoides; ns, differences not statistically significant; SS, Sézary syndrome; TE, terminal effector; T-LGLL, T-large granular lymphocytic leukemia; TM, transitional memory; T-PLL, T-prolymphocytic leukemia.

**SUPPLEMENTARY BIBLIOGRAPHY**

1. Botafogo, V., Pérez-Andres, M., Jara-Acevedo, M., Bárcena, P., Grigore, G., Hernández-Delgado, A., Damasceno, D., Comans, S., Blanco, E., Romero, A., *et al.* (2020). Age Distribution of Multiple Functionally Relevant Subsets of CD4+ T Cells in Human Blood Using a Standardized and Validated 14-Color EuroFlow Immune Monitoring Tube. Front. Immunol. *11*. 10.3389/FIMMU.2020.00166.

2. Pérez-Pons, A., Teodosio, C., Jara-Acevedo, M., Henriques, A., Navarro-Navarro, P., García-Montero, A.C., Álvarez-Twose, I., Lecrevisse, Q., Fluxa, R., Sánchez-Muñoz, L., *et al.* (2024). T-cell immune profile in blood of systemic mastocytosis: Association with disease features. Allergy Eur. J. Allergy Clin. Immunol. *79*, 1921–1937. 10.1111/all.16043.

3. Muñoz-García, N., Lima, M., Villamor, N., Morán-Plata, F.J., Barrena, S., Mateos, S., Caldas, C., Balanzategui, A., Alcoceba, M., Domínguez, A., *et al.* (2021). Anti-TRBC1 antibody-based flow cytometric detection of t-cell clonality: Standardization of sample preparation and diagnostic implementation. Cancers (Basel). *13*, 1–19. 10.3390/cancers13174379.

4. Langerak, A.W., Groenen, P.J.T.A., Brüggemann, M., Beldjord, K., Bellan, C., Bonello, L., Boone, E., Carter, G.I., Catherwood, M., Davi, F., *et al.* (2012). EuroClonality/BIOMED-2 guidelines for interpretation and reporting of Ig/TCR clonality testing in suspected lymphoproliferations. Leukemia *26*, 2159–2171. 10.1038/leu.2012.246.

5. Muñoz-García, N., Jara-Acevedo, M., Caldas, C., Bárcena, P., López, A., Puig, N., Alcoceba, M., Fernández, P., Villamor, N., Flores-Montero, J.A., *et al.* (2020). STAT3 and STAT5B Mutations in T/NK-Cell Chronic Lymphoproliferative Disorders of Large Granular Lymphocytes (LGL): Association with Disease Features. Cancers (Basel). *12*, 3508. 10.3390/cancers12123508.

6. Alaggio, R., Amador, C., Anagnostopoulos, I., Attygalle, A.D., Araujo, I.B. de O., Berti, E., Bhagat, G., Borges, A.M., Boyer, D., Calaminici, M., *et al.* (2022). The 5th edition of the World Health Organization Classification of Haematolymphoid Tumours: Lymphoid Neoplasms. Leukemia *36*, 1720–1748. 10.1038/S41375-022-01620-2.

7. Campo, E., Jaffe, E.S., Cook, J.R., Quintanilla-Martinez, L., Swerdlow, S.H., Anderson, K.C., Brousset, P., Cerroni, L., de Leval, L., Dirnhofer, S., *et al.* (2022). The International Consensus Classification of Mature Lymphoid Neoplasms: a report from the Clinical Advisory Committee. Blood *140*, 1229–1253. 10.1182/BLOOD.2022015851.

8. Gaillard, J.B., Chapiro, E., Daudignon, A., Nadal, N., Penther, D., Chauzeix, J., Nguyen-Khac, F., Veronese, L., and Lefebvre, C. (2023). Cytogenetics in the management of mature T-cell and NK-cell neoplasms: Guidelines from the groupe francophone de cytogénétique hématologique (GFCH). Curr. Res. Transl. Med. *71*. 10.1016/j.retram.2023.103428.

9. Van Dongen, J., Lhermitte, L., Bö Ttcher, S., Almeida, J., Van Der Velden, V., Flores-Montero, J., Rawstron, A., Asnafi, V., Lé Crevisse, Q., Lucio, P., *et al.* EuroFlow antibody panels for standardized n-dimensional flow cytometric immunophenotyping of normal, reactive and malignant leukocytes. 10.1038/leu.2012.120.
